# Rational engineering of the *Arabidopsis thaliana* plant cysteine oxidase 4 active site can reduce biochemical activity and improve submergence tolerance

**DOI:** 10.1016/j.jbc.2026.113331

**Published:** 2026-07-13

**Authors:** Anna Dirr, Arianna Del Greco, Simon A. Howe, Leah S. Walker, Monica Perri, Vinay Shukla, Francesco Licausi, Emily Flashman

**Affiliations:** 1 Department of Chemistry, University of Oxford, Oxford, United Kingdom; 2 Department of Biology, University of Oxford, Oxford, United Kingdom; 3 Biology Department, University of Pisa, Pisa, Italy

**Keywords:** Arabidopsis, dioxygenase, enzyme kinetics, hypoxia, oxygen sensing, plant cysteine oxidase, protein engineering, stress response

## Abstract

The increase in flooding events due to climate change is having devastating effects on food security. Plants can respond to submergence by reducing their energy requirements and switching their metabolism from oxidative phosphorylation to fermentation. This response is promoted by group VII ethylene response factors (ERF-VIIs) whose stability is dependent on oxygen availability; in normoxia, plant cysteine oxidase (PCO) enzymes catalyze oxidation of ERF-VII N-terminal Cys residues to trigger ERF-VII degradation *via* the Cys/Arg-N-degron pathway. In hypoxia, reduced PCO activity prevents ERF-VII degradation, enabling acclimation to anaerobic conditions. We have proposed previously that reducing PCO activity by enzyme engineering could enhance ERF-VII stabilization and improve submergence tolerance. Here, we describe the rational design and detailed *in vitro* analysis of a range of PCO variants based on the most active PCO isoform in *Arabidopsis thaliana*, *At*PCO4. We introduced single substitutions of specific amino acids in the active site and analysed their impact on enzyme catalysis, substrate affinity and iron binding capacity. We selected two variants with minimally or severely reduced activity (C173A and Y183F, respectively) and investigated their effect in an Arabidopsis model. Following submergence, survival and recovery were both improved in plants expressing the *At*PCO4 variants compared to *At*PCO4 wildtype. This proof-of-concept study demonstrates the potential to engineer just one amino acid in PCOs to improve flood resilience in plants and has implications for enhancing submergence tolerance in agronomically important crop species.

Floods are becoming more frequent due to extreme weather events. While some agronomical plants (such as rice and some wheat cultivars) can tolerate waterlogged roots and submerged aerial parts, many commercially relevant crop plants are less tolerant, including barley, maize, and soybean (1, 2). From 1991 to 2023 floods caused losses of USD 1.5 trillion, the biggest impact on agricultural profit from natural disaster (3). Flooded plants are severely affected by reduced O_2_ availability (hypoxia) caused by the slower diffusion of gases and impaired photosynthesis in the (often murky) flood water. This results in a carbohydrate and energy crisis eventually leading to plant death (reviewed in (4, 5, 6)).

Seed plants (spermatophytes) can detect and temporarily survive flooding *via* mechanisms regulated by the Cys/Arg-N-degron pathway (Fig. S1) that support a shift to anaerobic metabolism to maintain basal levels of ATP production (7, 8, 9). Under normoxic conditions the N-terminal Met is cleaved from protein substrates by methionine aminopeptidases (MAPs) exposing an N-terminal Cys (Nt-Cys) (10). Plant cysteine oxidases (PCOs) then catalyze the dioxygenation of the Nt-Cys of the protein substrate to cysteine sulfinic acid (Nt-Cys-SO_2_H) which is recognized by arginyl transferases (ATEs) (11, 12). ATEs catalyze the transfer of an Arg onto the respective protein substrate N-terminus (12), labelling it for ubiquitination by the E3 ligase proteolysis 6 (PRT6) and proteasomal degradation (Fig. S1) (13, 14). To date, seven protein substrates of the Cys/Arg-N-degron pathway have been identified in plants; five of them belong to group VII ethylene response factors (ERF-VIIs) responsible for inducing the transcription of hypoxia responsive genes (HRGs) (7, 8) and the remaining two, vernalization 2 (VRN2) (15) and little zipper 2 (ZPR2) (16), are involved in developmental processes.

The rate-limiting step of the Cys/Arg-N-degron pathway is the dioxygenation of Nt-Cys substrates catalyzed by PCOs which sense the availability of O_2_ (11, 17). When O_2_ levels decrease (causing hypoxia) in a submerged plant, PCOs are catalytically less active leading to the stabilization of their substrates, including ERF-VIIs (Fig. S1) (7, 8, 11). Once stabilized, ERF-VIIs induce the transcription of HRGs including alcohol dehydrogenase 1 (*ADH1*), pyruvate decarboxylase 1 (*PDC1*) and sucrose synthase 4 (*SUS4*) (7, 8, 9, 18). ERF-VII accumulation and function is important both during submergence and upon de-submergence and, in addition to oxidation by PCOs, can be impacted by other signals (comprehensively described elsewhere) (2, 5, 19, 20). Increased accumulation of endogenous ERF-VIIs, if transient, has been shown to be beneficial for submergence survival of Arabidopsis plants, with similar outcomes reported in barley and maize (7, 21, 22, 23). However constitutive expression of a stabilized ERF-VII in Arabidopsis resulted in poorer aerobic growth and recovery from submergence (7). We have previously shown that mutations which inactivate *At*PCO4 result in plants with stunted growth and infertility, likely due to constitutive ERF-VII stability and HRG expression (18). We have therefore proposed that dynamic regulation of the ERF-VIIs to allow prolonged stabilization or increased accumulation during submergence could be achieved by reducing the rate of PCO activity (24). In this study, we have mutated single residues in the active site of *At*PCO4, the most active of the PCO enzymes in Arabidopsis, aiming to slow its activity and thus reduce the rate at which ERF-VIIs are degraded (9, 11, 17).

The crystal structures of *At*PCO4, *At*PCO5 and *At*PCO2 reveal a double-stranded beta-helix (DSBH) core categorizing PCOs as members of the cupin fold superfamily. The active site lies within the cupin fold and is characterized by an Fe(II) cofactor that is coordinated in octahedral geometry by three His residues and three water molecules in non-substrate bound structures (Fig. 1). The His-triad is characteristic for thiol dioxygenases, distinguishing them from other oxygenases (18, 25, 26). Thiol dioxygenases can be further classified based on their substrate preferences into small molecule thiol dioxygenases (smTDOs), such as the human cysteine dioxygenase (*Hs*CDO), and Nt-Cys thiol dioxygenases (NCOs), such as PCOs and the human 2-aminethanethiol dioxygenase (*Hs*ADO) (25). We compared the active site of *At*PCO4 to *Hs*ADO and *Hs*CDO to identify prospective residues for reducing *At*PCO4 activity (Fig. 1*B*). We anticipated that the active site of *Hs*CDO would be interesting for comparison due to the different substrate specifications and *Hs*ADO because of its higher O_2_ sensitivity (*K*_*M*_(O_2_) ∼ 47–55% O_2_) (27) compared to PCOs (*At*PCO4 *K*_*M*_(O_2_) ∼ 17% O_2_) (17, 28), a trait which might be beneficial in reducing PCO activity. Also, despite both being NCOs, the substrate sequence downstream of the Nt-Cys differs between PCO and ADO substrates, *for example*, ERF-VIIs (CGGAI/VISD) (17) compared to regulator of G protein signaling four/five (RGS4/5: CKGLAG/ALP) (27). We used these comparisons as a foundation for our structure/function-guided approach to modify *At*PCO4 *in vitro* to learn more about the influences of particular residues on *At*PCO4 activity and engineer an *At*PCO4 with reduced but not ablated activity. In addition to modifying *At*PCO4 activity *in vitro*, we wanted to test whether this approach is applicable *in planta* and can increase submergence tolerance. In this study, we therefore introduced wildtype (WT) or variant *AtPCO4* sequences into *4pco* Arabidopsis plants; this model allows assessment of the effect of *AtPCO4* mutations on phenotype and submergence resilience without redundancy effects from other *At*PCO enzymes (18).

**Figure 1:**
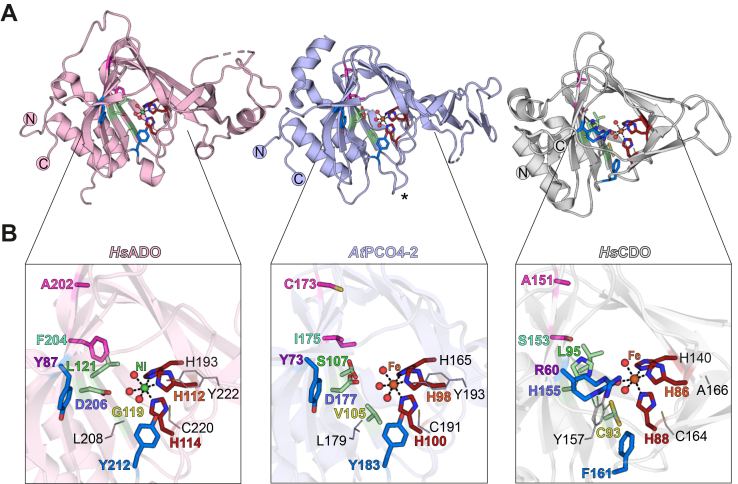
**Structural comparison of *At*PCO4 with the thiol dioxygenases *Hs*ADO.***A*, cartoon representation of crystal structures of *Hs*ADO (PDB: 7REI), *At*PCO4 (PDB: 6S7E, structure shown is *At*PCO4-1 but residues numbered according to *At*PCO4-2 to align with main text) and *Hs*CDO-1 (PDB: 6CDH). Asterix indicates the proposed substrate binding loop (residues 183–190). *B*, cartoon representation of the active site of each enzyme with residues coloured according to their location (*red*: iron coordinating His-triad; *blue*: residues at potential substrate entry site; *pink*: residues *trans* to substrate entry site; *green*: residues *trans* to His-triad).

Targeted mutagenesis of specific residues within three regions of the active site that are important for *At*PCO4 activity (Fig. 1) revealed the opportunity to lower *At*PCO4 activity by reducing substrate affinity or catalytic rate. Following biochemical and kinetic characterization, we selected two *At*PCO4 variants, *At*PCO4 C173A and Y183F, to complement the Arabidopsis *4pco* model and test their effect on submergence tolerance *in vivo*. Plants containing either of these *At*PCO4 variants displayed improved submergence resilience compared to WT controls. Thus, our work supports the principle that engineering PCO function is a viable route to improve submergence tolerance in plants, and that this can be achieved with single amino acid substitutions.

## Results

### Selection of *At*PCO4 isoform for variant generation

Before designing *At*PCO4 variants, we wanted to ensure we were using the appropriate *AtPCO4* WT sequence for the Arabidopsis Columbia-0 (Col-0) plants in our laboratory because *At*PCO4 can be expressed as two splice variants, *At*PCO4-1 and *At*PCO4-2. *At*PCO4-2 contains an additional Glu residue at position 135 (E135) that is located in a loop away from the active site (Fig. S2*A*). Col-0 plants in our laboratory showed higher transcription levels of *AtPCO4-2* (around 70%) than *AtPCO4-1* (around 30%). Since previous *in vitro* data focused on *At*PCO4-1 (18), we examined whether there are differences in *At*PCO-1 and *At*PCO4-2 activities *in vitro*, using previously reported methodology (17, 29). We did not observe any significant difference in the activity of the two *At*PCO4 isoforms (Fig. S2) allowing the comparison of *in vitro* data from *At*PCO4-2 with *At*PCO4-1. *At*PCO4-2 was therefore used in this study to investigate active site residues *in vitro* and in the *4pco* Arabidopsis model to align with higher transcription of *AtPCO4-2* in our Col-0 plants. Herein, *At*PCO4-2 will be referred to as *At*PCO4.

### Replacement of iron-coordinating residues ablates *At*PCO4 activity

All *At*PCOs contain a conserved iron coordinating His-triad (Fig. 1) (18, 26). This is in contrast to other non-heme Fe(II) dioxygenases, including the metazoan oxygen-sensing enzyme hypoxia-inducible factor (HIF) hydroxylases (30), which typically coordinate Fe(II) with a His/Asp/His-motif (25, 31). We and others have previously replaced *At*PCO4(-1) His164 with an Asp to mimic this motif, however this completely abolished *At*PCO4 activity (18, 26). To test whether similar replacement of the other His-triad residues in *At*PCO4 with an Asp (*At*PCO4 H98D and H100D) also resulted in enzyme inactivation, we generated recombinant forms of these variants and tested their capability to catalyze oxidation of RAP2.12_2-15_, an Nt-Cys2 initiating peptidic form of an ERF-VII substrate. As anticipated, these mutations also resulted in abolished activity of *At*PCO4 enzymes, likely connected, at least in part, to their reduced iron retention capability (Fig. S3 (32)) which was in line with previous data (18). Similar results have been reported for replacing the His-triad residues with an Asp in *Hs*CDO-1 in density functional theory studies (33). As expected, all residues of the His-triad appear to be essential for the efficient catalysis of Cys oxidation by thiol dioxygenases.

### Residues involved in substrate binding reduce *At*PCO4 activity by decreasing enzyme affinity for peptide substrates

Based on studies in other thiol dioxygenases, it is considered likely that PCO enzymes coordinate substrates *via* the Nt-Cys amino and thiol groups at the active site Fe(II), prior to O_2_ binding and activation, allowing Nt-Cys oxidation *via* a Fe(III)-superoxo or Fe(IV)-oxo intermediate (25, 34). To date, no substrate-bound PCO structure has been reported. However, models of PCO (18, 26) and the mammalian thiol dioxygenase ADO (35, 36, 37) in complex with substrates, as well as structures of enzyme-cyclic peptide inhibitor complexes (34) indicate that substrate binds in the previously predicted region of the enzyme (highlighted with an asterix in Fig. 1*A*) (25). Comparison of *At*PCO4 with the structure of *Hs*ADO, which also catalyzes oxidation of the Nt-Cys of target proteins (27), shows that residues Y73 and Y183 are conserved at the potential substrate entry site (Fig. 1*B*, residues in blue); modelled structures of *At*PCO4 docked with peptide substrates suggest that these residues might be important for correct positioning of Nt-Cys substrates (Fig. S4 (15, 16, 32, 34, 37, 38, 39)). Comparison of *At*PCO4 with the structure of *Hs*CDO, which catalyzes oxidation of free l-cysteine, reveals that the equivalent residues to Y73 and Y183 are R60 and F161, respectively (Fig. 1). In previous work investigating the active site residues of *At*PCO4, we reported reduced (but not abolished) activity for a Tyr to Phe variant, *At*PCO4(-1) Y182F (18). We therefore hypothesized that this and other mutations in this region may decrease PCO activity through reduced substrate binding. This has been confirmed recently for at least the Tyr in *Hs*ADO (34) corresponding to Y183 in *At*PCO4. We therefore generated and kinetically investigated the *At*PCO4 Y183F variant (equivalent to the *At*PCO4(-1) Y182F variant previously reported (18)) to determine the importance of the Tyr-OH group in substrate binding.

Residue Y73 is positioned further away from the active site and shows flexible movement in docking models depending on substrate (Figs. S4 and S5*B*). In *Hs*ADO the equivalent Tyr residue is predicted to form an H-bond with its substrate (Fig. S5*A* (32)) in a docking model and, although this is not predicted for the *At*PCO4-RAP2.12_2-7_ interaction (Fig. S5B (32)), we hypothesized that a significant change in structure of the residue at this position may disrupt substrate binding. A recent study revealed that mutating the equivalent Tyr residue in *Hs*ADO to an Ala or Phe did reduce activity by ∼30 to 70% but did not considerably alter the enzyme’s substrate affinity (34). To interrupt substrate affinity more profoundly than with a Tyr to Phe substitution, we designed an *At*PCO4 Y73R variant; given the presence of R60 in a structurally equivalent position in *Hs* CDO (Fig. 1), we hypothesized that such a mutation could be structurally tolerated.

**Figure 2:**
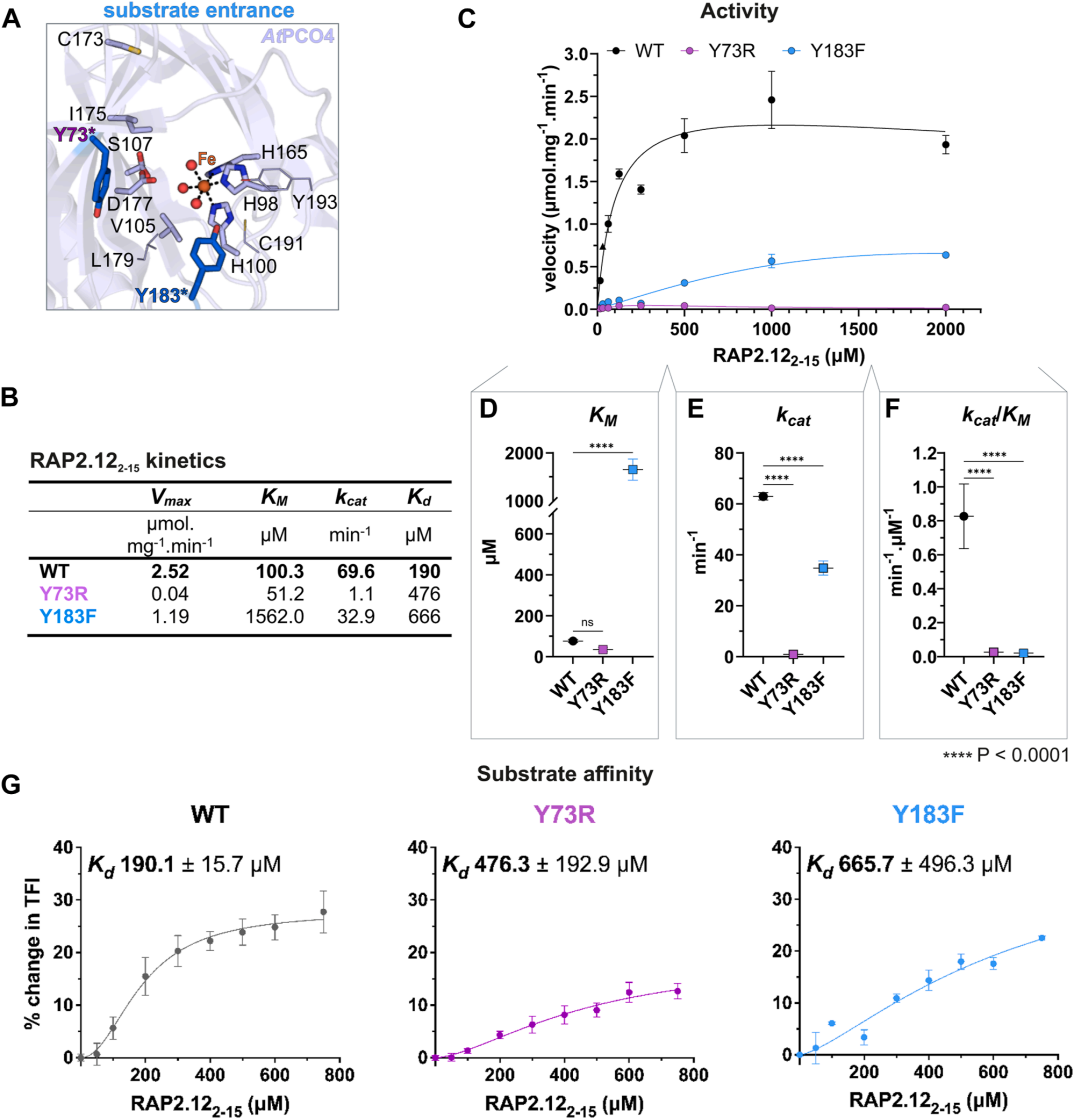
**Activity and substrate binding of substrate entry site variants.***A*, cartoon representation of *At*PCO4 crystal structure (PDB: 6S7E, crystal structure of *At*PCO4-1 but residues were numbered according to *At*PCO4-2 to align with main text) with investigated residues highlighted in *bright blue* and with asterix. *B*, Kinetic parameters derived for *At*PCO4 enzymes with substrate peptide. Parameters for Y183F were determined based on additional data shown in the supplementary (Fig. S10*C*). *C*, Initial velocities of *At*PCO4 WT (from two independent assays in technical triplicates, 2 × n = 3) and variants (in technical triplicates, n = 3) from 0 to 2 min time courses plotted against peptide substrate concentrations. Values are mean ± standard deviation (as indicated by error bars). *D – F*, graphic overview of (*D*) Michaelis-Menten constant, (*E*) catalytic constant and (*F*) catalytic efficiency of *At*PCO4 WT and variant values in (*B*) calculated using the Michaelis-Menten equation in GraphPad Prism, Version 10.1.2. Values are mean ± standard error (as indicated by error bars). The statistical significance (*p* < 0.05) of the kinetic constants from each variant in comparison to *At*PCO4 WT were evaluated using one-way ANOVA followed by Holm-Šídák's test to correct for multiple comparisons using hypothesis testing (GraphPad Prism). *G*, affinity of RAP2.12_2–15_ to *At*PCO4 WT (n = 6) and variants (n = 3) determined using an intrinsic fluorescence tryptophan assay (39). A specific binding model with Hill slope (Y = *B*_*max*_∗X^h^∗(*K*_*d*_^*h*^ + X^h^)^−1^) was fitted to the data with error bars displaying the standard error (GraphPad Prism). The increased error values for variants, Y73R and Y183F, likely stem from the reduced binding affinity of both variants. To reach saturating conditions, higher substrate concentrations are required but this is limited technically due to peptide solubility. The % change in tryptophan fluorescence intensity (TFI) was lower in *At*PCO4 Y73R compared to the WT enzyme which might reflect a change in the microenvironment of the Trp residue due to mutation Y73R. However, overall secondary structure elements of the variant were similar to WT enzyme (Fig. S7).

Recombinant *At*PCO4 variants Y73R and Y183F were successfully produced (Figs. S6–S8) and assay conditions were systematically adjusted for optimal activity. including optimization of supplemental Fe(II) for each enzyme (Fig. S9) (29). We opted to measure (here and throughout this study) enzyme activity per μg total protein, rather than using iron occupancy to determine the proportion of active enzyme, so that overall enzyme activity reflected any impact of the mutations on iron retention. Kinetic assays revealed that both Y73R and Y183F variants were significantly less active towards the peptide substrate RAP2.12_2-15_ than the WT enzyme (Fig. 2, *B*–*F*) with significantly reduced catalytic turnover (*k*_*cat*_ of WT 69.6 min^−1^, Y73R 1.1 min^−1^ and Y183F 32.9 min^−1^) and catalytic efficiency (*k*_*cat*_/*K*_*M*_ of WT 0.69 min^−1^ μM^−1^, Y73R and Y183F 0.02 min^−1^ μM^−1^, Fig. 2, *D*, *F* and *G*, Table S5). We proposed that these mutations would reduce substrate binding, therefore used a tryptophan fluorescence intensity (TFI)-based assay to measure affinity of the enzymes for substrate (39). The results revealed that the decrease in activity could be ascribed to a considerably weakened interaction between the variants and RAP2.12_2-15_ (Fig. 2*G*, *K*_*d*_ of WT 190 μM, Y73R 476 μM and Y183F 666 μM). We used circular dichroism and inductively coupled plasma mass spectrometry (ICP-MS) to confirm that the reduced substrate binding of the variants was neither due to major structural changes (Figs. S7 and S8) nor due to reduced iron binding (iron occupancy of WT 17%, Y73R 33% and Y183F 50%; Fig. S10*B*). Overall, the data indicate that *At*PCO4 residues Y73 and Y183 play an important role in substrate binding and as a consequence impact effective catalysis. While removal of the Y183-OH group resulted in reduced *At*PCO4 activity, the substitution Y73R resulted in an almost complete loss of enzymatic activity.

### Residues close to active site Fe(II) alter catalysis independent of overall substrate affinity

We next wanted to explore the effects of mutating residues that might have a direct role in *At*PCO4 catalysis *via* interaction with the Nt-Cys, O_2_ and/or reaction intermediates, focusing on D177, V105 and S107 which are on the opposite face (trans) of the active site Fe(II) from the His-triad. The carboxylate group of *At*PCO4 residue D177 has been proposed to stabilize the Nt-Cys amine or thiol of protein substrates (D176 in *At*PCO4-1, Fig. S4 (32)) (18, 26) or it could influence interaction with O_2_ during turnover. Both interactions have been suggested for the equivalent Asp in mammalian ADO (D206 in *Hs*ADO) (34, 35, 37). Removing the negative charge of the Asp residue by replacing it with an Asn (*At*PCO4(-1) D176N) has previously been shown to significantly reduce *At*PCO4 activity (18). We therefore reasoned that substituting the Asp with a Glu (D177E) could still reduce *At*PCO4 activity but to a lesser degree than the previous Asp to Asn substitution (18) because the negative charge would be maintained. During the preparation of this manuscript the equivalent Asp to Glu mutation was examined in the *Hs* ADO resulting in ∼20% activity compared to WT while Asp to Asn mutation resulted in ∼2% activity (34). *At*PCO4 residues V105 and S107 are in a structurally homologous position to *Hs*ADO residues G119 and L121, respectively (Fig. 1). We therefore prepared *At*PCO4 variants V105G and S107L, reasoning that they should be structurally tolerated by the enzyme and hypothesizing that they might affect the positioning, and therefore stability, of O_2_ and Nt-Cys substrate or reaction intermediates and thus impact their kinetic properties.

**Figure 3:**
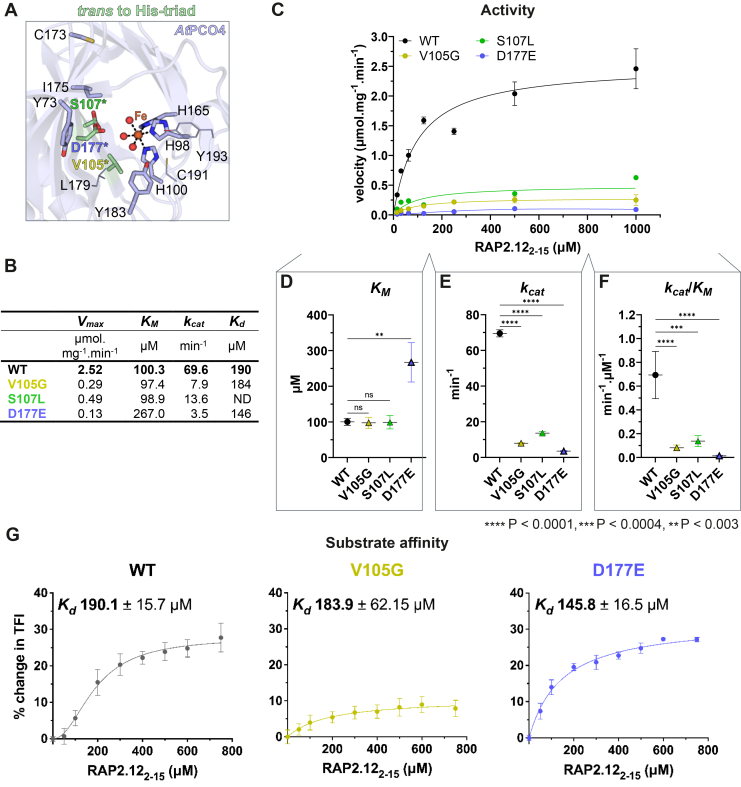
**Activity and substrate binding of variants with mutations approximately *trans* to the His-triad.***A*, cartoon presentation of *At*PCO4 crystal structure (PDB: 6S7E, crystal structure of *At*PCO4-1 but residues were numbered according to *At*PCO4-2 to align with main text) with investigated residues highlighted in *green* and with asterix. *B*, Values of the Michaelis-Menten kinetics for *At*PCO4 enzymes with substrate peptide. *C*, Initial velocities of *At*PCO4 WT (from two independent assays in technical triplicates, two x n = 3) and variants (in technical triplicates, n = 3) from 0 to 2 min time courses plotted over peptide substrate concentrations. Values are mean ± standard deviation (as indicated by error bars). *D – F*, graphic overview of (*D*) Michaelis-Menten constant, (*E*) catalytic constant and (*F*) catalytic efficiency of *At*PCO4 WT and variants calculated using the Michaelis-Menten equation in GraphPad Prism. Values are mean ± standard error (as indicated by error bars). The statistical significance (*p* < 0.05) of the kinetic constants from each variant in comparison to *At*PCO4 WT were evaluated using one-way ANOVA followed by Holm-Šídák's test to correct for multiple comparisons using hypothesis testing (GraphPad Prism). Only significant differences to *At*PCO4 WT are displayed. *G*, affinity of RAP2.12_2–15_ to *At*PCO4 enzymes determined using an intrinsic fluorescence tryptophan assay (39). The *K*_*d*_ was estimated using the one-site (V105G with n = 6, D177E with n = 3; Y = *B*_*max*_∗X∗(*K*_*d*_ + X)^−1^) or Hill-slope (WT with n = 6) specific binding model in GraphPad Prism (technical repeats). The % change in tryptophan fluorescence intensity (TFI) was lower in *At*PCO4 V105G compared to the WT enzyme which might reflect a change in the microenvironment of the Trp residue due to mutation V105G that did not affect substrate binding.

We produced recombinant *At*PCO4 variants D177E, V105G and S107L, checked the integrity of their secondary structures (Figs. S6–S8) and optimized assay conditions for each enzyme, including supplemental Fe(II) concentration (Fig. S9). CD analysis suggested that insertion of the D177E mutation perturbed the proportions of α-helix and β-sheet in the overall protein fold in this variant, although this effect was not observed when D177E was reconstituted with Ni(II) (Figs. S8 and S9). Kinetic analysis revealed that all three mutations decreased *At*PCO4 activity compared to WT enzyme, demonstrated by significantly lower *k*_*cat*_ (WT 69.6 min^−1^, V105G 7.9 min^−1^, S107L 13.6 min^−1^ and D177E 3.5 min^−1^) and catalytic efficiency values (WT 0.69 min^−1^ μM^−1^, V105G 0.08 min^−1^ μM^−1^, S107L 0.14 min^−1^ μM^−1^ and D177E 0.01 min^−1^ μM^−1^; Fig. 3, *B*–*F*, Table S5). Interestingly, variants V105G and S107L showed similar *K*_*M*_ values to WT (*K*_*M*_ of WT 100.3 μM, V105G 97.4 μM and S107L 98.9 μM; Fig. 3, *B* and *D*), indicative that these mutations did not affect the stability of the enzyme–substrate complex. Substrate binding affinity was determined for the V105G variant, and although CD data suggested that the introduction of the Gly residue in this position may have decreased stability of the enzyme during preparation of the variant for the binding assay (requiring removal of Fe(II) and substitution with Ni(II) (39), Fig. S8), the determined *K*_*d*_(V105G) of ∼183.9 ± 62 μM, was similar to *K*_*d*_(WT) 190 ± 16 μM (Fig. 3*G*). The lower activity observed for variant S107L may be attributed to the lower iron occupation measured for this variant, despite addition of higher amounts of exogenous Fe(II) to kinetic assays (Fig. S11 (32, 40)). Variant D177E retained its iron-binding ability (Fig. S11) and had a slightly reduced *K*_*d*_ value compared to the WT enzyme (*K*_*d*_ of WT 190.1 ± 16 μM and D177E 145.8 ± 17 μM; Fig. 3*G*) suggesting similar or higher affinity of variant D177E to RAP2.12_2-15_ substrate. The *K*_*M*_ value of variant D177E (267 μM) was likely high (relative to the WT) due to the low activity (*k*_*cat*_) of this variant (Fig. 3, *B* and *E*). *In silico* docking models indicated that a Glu residue at position 177 can either coordinate the iron or the substrate (Fig. S11), both of which could affect the positioning of the iron and the substrate in the active site, consistent with the D177E mutation altering the rate of catalysis through altered stability of intermediates rather than reducing substrate or iron binding.

**Figure 4:**
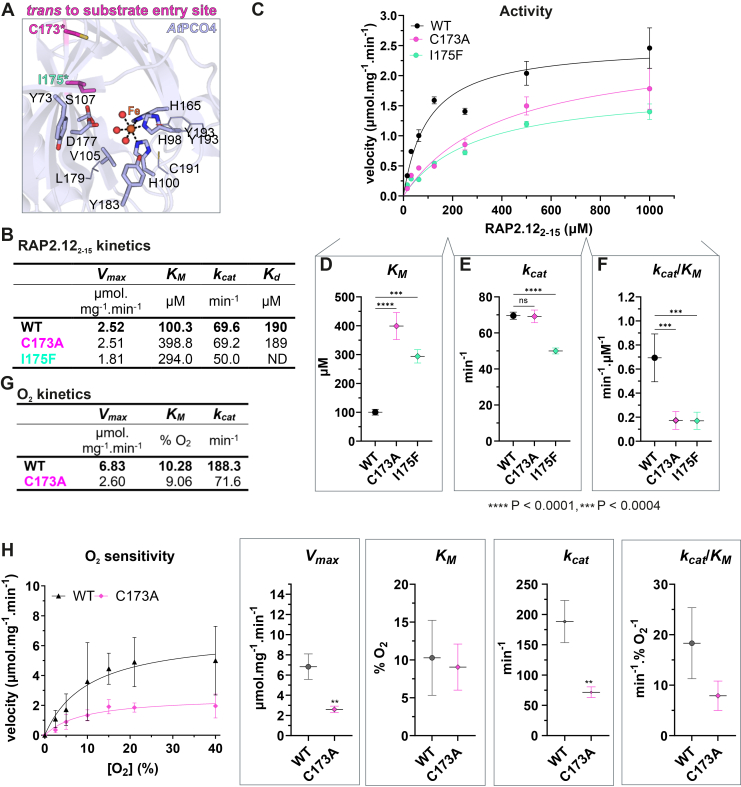
**Activity and substrate binding of residues affecting *At*PCO4 catalysis and being located approximately *trans* to the substrate entry site.***A*, cartoon presentation of *At*PCO4 crystal structure (PDB: 6S7E, crystal structure of *At*PCO4-1 but residues were numbered according to *At*PCO4-2 to align with main text) with investigated residues highlighted in magenta and with asterix. *B*, Values of the Michaelis-Menten kinetics for *At*PCO4 enzymes with substrate peptide. *C*, initial velocities of *At*PCO4 WT (from two independent assays in technical triplicates, 2 × n = 3) and variants (in technical triplicates, n = 3) from 0 to 2 min time courses plotted over peptide substrate concentrations. Values are mean ± standard deviation (as indicated by error bars). *D–F*, graphic overview of mean of (*D*) Michaelis-Menten constant, (*E*) catalytic constant, and (*F*) catalytic efficiency of *At*PCO4 WT and variants determined using the Michaelis-Menten equation in GraphPad Prism. Values are mean ± standard error (as indicated by error bars). The statistical significance (*p* < 0.05) of the kinetic constants from each variant in comparison to *At*PCO4 WT were evaluated using one-way ANOVA followed by Holm-Šídák’s test to correct for multiple comparisons using hypothesis testing (GraphPad Prism). *G–H*, O_2_ sensitivity of AtPCO4 WT and variant C173A (*H*) measured after 1 min reaction time (*left*, replicates: 2 × n = 3). Values are mean ± standard deviation (as indicated by error bars). Michaelis-Menten constants were estimated and statistical significance (*p* < 0.05) evaluated using Welch’s *t* test (∗∗: *p* = 0.0024, GraphPad Prism). Error bars indicate standard error of the mean. Only significant differences to *At*PCO4 WT are displayed.

We next investigated residues C173 and I175, which are positioned approximately *trans* to the substrate entry site. We hypothesized that they may play a role in stabilizing Fe-oxygen intermediates due to their location within the active site, albeit they are distant from direct interactions with substrate (Fig. 4*A*, residues in magenta). Interestingly, residue C173 appears to be conserved in all PCOs but not in *Hs*ADO nor *Hs*CDO-1 which both have an Ala residue at this position (A202 in *Hs*ADO and A151 in *Hs*CDO-1, Fig. 1*B*). Residue C173 was therefore mutated to an Ala. The equivalent residue to *At*PCO4 I175 in *Hs*ADO is a Phe (Fig. 1*B*, residues in magenta). The bulkier Phe in *Hs*ADO might restrict movement of O_2_ around the iron and, therefore, also O_2_ activation; *At*PCO4 I175 was therefore mutated to a Phe. We reasoned that both variants could be structurally tolerated but hypothesized that they may both demonstrate reduced activity through reduced stability of reaction intermediates. Recombinant *At*PCO4 variants C173A and I175F were successfully produced, their correct folding was confirmed by CD spectroscopy (Figs. S6–S8), assay conditions were optimized, including supplemental Fe(II) (Fig. S9) and their activities were kinetically analyzed.

*At*PCO4 I175F demonstrated reduced catalytic efficiency (*k*_*cat*_/*K*_*M*_ of I175F 0.17 min^−1^ μM^−1^ compared to WT 0.69 min^−1^ μM^−1^) by decreasing the catalytic turnover rate (*k*_*cat*_ of I175F 50.0 min^−1^ compared to WT 69.6 min^−1^) and increasing the *K*_*M*_ of *At*PCO4 (*K*_*M*_ of I175F 294 μM compared to WT 100.3 μM; Fig. 4, *B*–*F*, Table S5). Variant C173A demonstrated a *k*_*cat*_ value similar to the WT enzyme (*k*_*cat*_ 69.2 min^−1^), while its catalytic efficiency was decreased (*k*_*cat*_/*K*_*M*_(WT) 0.69 min^−1^ μM^−1^, *k*_*cat*_/*K*_*M*_(C173A) 0.17 min^−1^ μM^−1^) owing to a higher *K*_*M*_ value (*K*_*M*_(WT) ∼100 μM, *K*_*M*_(C173A) ∼400 μM; Fig. 4, *B*–*F*). Neither mutation reduced the iron occupancy of the enzyme (Fig. S12), in fact both showed higher iron occupancy than WT.

The location of residues C173 and I175 approximately *trans* to the substrate entry site and > 6.5 Å distant from the Nt-Cys substrate (Fig. S12 (32, 40)) led us to hypothesize that the higher *K*_*M*_ values observed for these variants may reflect different requirements for the O_2_ co-substrate rather than the peptide substrate RAP2.12_2-15_. Focusing on the C173A variant, due to its apparent similar *k*_*cat*_ to *At*PCO4 WT (Fig. 4, *B* and *E*), we therefore determined the kinetic parameters of this variant with respect to O_2_, under conditions of optimal RAP2.12_2-15_ availability. Measuring the activity of *At*PCO4 C173A at 0 to 40% O_2_ with 1500 μM RAP2.12_2-15_ revealed that variant C173A could not use O_2_ as efficiently as the WT enzyme (Fig. 4*H*). This was represented by a *K*_*M*_(O_2_) similar to the WT enzyme (*K*_*M*_(O_2_) of WT 10.28% and C173A 9.06%) but lower *k*_*cat*_ values (*k*_*cat*_(O_2_) of WT 188.3 min^−1^ and C173A 71.6 min^−1^; Fig. 4, *G* and *H*). We noted that in the O_2_ assay, WT enzyme was able to achieve a greater *V*_*max*_ than in the RAP2.12_2-15_ assay, potentially due to limiting O_2_ in normoxic experiments as well as differences in assay conditions (Fig. S12*D*). To confirm that mutating a residue that is located approximately *trans* to the substrate entry site mainly affects the catalysis of the dioxygenation of substrate peptide and not peptide substrate binding, the *K*_*d*_ of variant C173A was determined and found to be similar to the WT enzyme (*K*_*d*_(WT) ∼190 ± 16 μM, *K*_*d*_(C173A) 189.4 ± 57 μM, Fig. S12, *E* and *F*). Also, *in silico* docking models showed Nt-Cys positioning in variant C173A similar to that in the WT enzyme (Fig. S12*A* (32, 34, 37, 40)). Overall, although C173A was the most active of all the examined variants, it was observed to catalyze the dioxygenation of RAP2.12_2-15_ peptide less efficiently than the WT enzyme; this mutation seemed to have a rate-limiting impact on enzyme activity with respect to O_2_. The mechanistic role of C173 in controlling rates of reaction with O_2_ at the active site will be examined in future studies.

### Reduced *At*PCO4 activity correlates with increased submergence tolerance in *4pco Arabidopsis* plants

We have previously reported that *At*PCO4 variants with biochemically ablated activity also showed ablated activity *in vivo*, when introduced into the *4pco* Arabidopsis model (18). To improve submergence tolerance in plants, our strategy seeks to enhance ERF-VII stability and thereby function during a submergence event, through reduced *At*PCO activity, while maintaining sufficient *At*PCO-initiated degradation of ERF-VIIs under normoxic conditions such that the plant is not in a permanent metabolically “stressed” state. Having generated a range of activity-limiting *At*PCO4 variants, we selected two that had shown reduced or similar activity to *At*PCO4 WT *in vitro*, to introduce into Arabidopsis *4pco* plants and test their impact *in vivo*. We selected *At*PCO4 C173A as it was the most catalytically competent of all the variants in normoxia. We also selected variant Y183F to test the effect of significantly reduced substrate binding on *At*PCO4 function *in vivo*.

As previously reported, *4pco* plants, which lack all *At*PCOs except *At*PCO3, exhibit developmental defects that can be complemented with functional *At*PCO enzymes to establish a Col-0 like phenotype (9, 18, 27). We therefore established two lines per genotype for *4pco AtPCO4* WT (wt-1 and wt-2) and for *4pco AtPCO4-C173A* (C173A-1 and C173A-2); we were only able to establish one line for *4pco AtPCO4-Y183F* (Fig. S13, sequences in Figs. S14–S16 (41)). The complemented *4pco* lines had a phenotype similar to that of Col-0 plants (Fig. S17*C*, Tables S6–S8 (42)) although *4pco* lines C173A-1 and Y183F, had a lower germination rate and were slightly developmentally delayed (Fig. S17, *B*–*D* (42)). The similar phenotype of *4pco AtPCO4* variant plants to Col-0 is in contrast to the phenotype we have previously reported for *4pco* plants complemented with inactive *At*PCO variants (18) which showed a phenotype more similar to that of *4pco* plants (27).

**Figure 5:**
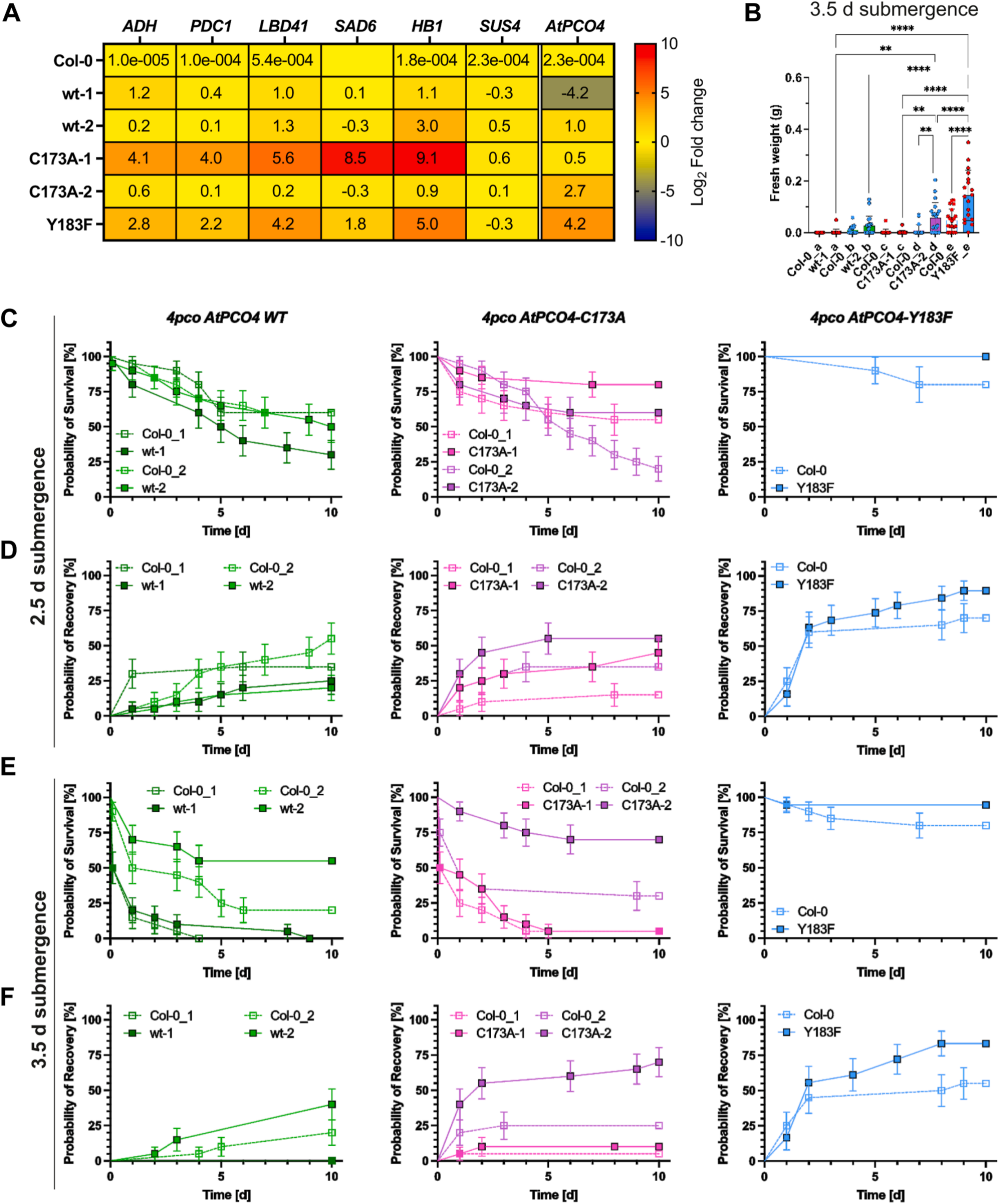
**Effect of *At*PCO4variantson submergence tolerance in the *4pco AtPCO4* Arabidopsis plant model.***A*, change in transcript levels of HRGs and *AtPCO4* WT and mutants (primer pair: qpAtPCO4-2) in 5.5-week-old *4pco AtPCO4* and Col-0 plants. Two independent experiments with two technical replicates of four biological samples each (n = 2 × (2 × 4)). *B*, fresh weight of plants submerged for 3.5 days after 10 days of recovery (lower case letters indicate plants submerged in the same box). Dead plants were considered to weigh 0 g. The statistical significance (*p* < 0.05) was determined using two-way ANOVA followed by Tukey's multiple comparisons test (GraphPad Prism). Only significant differences between *4pco AtPCO4* lines and their control Col-0 lines or other *4pco AtPCO4* lines are displayed (∗∗: *p* < 0.0084; ∗∗∗∗: *p* < 0.0001). *C and E*, survival of each *4pco AtPCO4* line (filled box, continuous line) and its Col-0 control (empty box, dashed line, submerged in same box) after (*C*) 2.5 or (*E*) 3.5 days submergence was scored using a dead/alive scoring system. To be considered alive, the plant needed to have a *green* meristem with at least the area of one leaf in a fresh *green* colour attached. Values represent two independent experiments with 10 plants/line/experiment (n = 2 × 10), except for line Y183F (n = 1 × 10 plants). *D and F*, recovery rate of each *4pco AtPCO4* line and its Col-0 control group after (*D*) 2.5 or (*F*) 3.5 days submergence is shown as described for (*C*) and (*E*). A plant was scored as recovering, when it produced a new leaf and that leaf continued to grow. Abbreviations: ADH: alcohol dehydrogenase, PDC1: pyruvate decarboxylase 1, LBD41: LOB domain-containing protein 41, SAD6: stearoyl-acyl Carrier Protein 9-Desaturase 6, HB1: plant haemoglobin 1, SUS4: sucrose synthase 4, *AtPCO4*: *AtPCO4* insert.

ERF-VII stability and consequent HRG expression can in principle be impacted by both PCO enzyme abundance and catalytic competency. Anti-PCO antibodies are not currently available to compare protein levels in transgenic plants, however we measured the transcript level of each introduced *AtPCO4* gene. Although *AtPCO4* WT and mutant genes were transformed into *4pco* plants together with the endogenous *AtPCO4* promotor (33 bp upstream of *AtPCO4* WT or mutant gene) to enable constitutive expression, their insert location into the Arabidopsis genome could not be controlled during transformation using Agrobacteria. We compared *AtPCO4* mRNA levels to the housekeeping gene polyubiquitin 10 (*UBQ10*), normalised to Col-0, and found that transcript levels varied amongst the different lines (Fig. 5*A*): wt-1 < Col-0 < C173A-1 < wt-2 < C173A-2 < Y183F (Figs. 5*A* and S18). The apparent downregulation of *AtPCO4* transcription in wt-1 was surprising since its phenotype was similar to that of Col-0 (Fig. S17 (42)). It is possible that the high catalytic efficiency of *At*PCO4 (17) results in similar levels of ERF-VII oxidation and therefore HRG transcript abundance to Col-0 despite lower expression levels of *At*PCO4.

We next examined the impact of the mutations on normoxic HRG transcript levels as an indirect measure of ERF-VII protein stability arising from decreased catalytic capability of the *At*PCO4 variants. We measured the levels of HRG transcripts in each of the *4pco AtPCO4* mutant lines relative to the housekeeping gene *UBQ10* (Fig. 5*A*). All of the tested marker genes for hypoxia responses (except *SUS4* encoding sucrose synthase 4) showed increased transcript levels for line C173A-1 and to a lesser degree for line Y183F, while their transcript levels in line C173A-2 were similar to wt-1, wt-2 and Col-0 (Figs. 5*A* and S19). Collectively, the increased HRG levels in the variant lines (when *AtPCO4* transcript levels were similar to WT) suggested that the *AtPCO4* mutations reduced the activity of *At*PCO4 *in vivo* leading to ERF-VII stabilization. We also observed that the effect of decreased *At*PCO4 activity could be, in part, neutralized by higher *AtPCO4* mutant transcription, as seen in the case of line C173A-1 vs line C173A-2 (Fig. 5*A*). The increased *ADH*, *PDC1* and *LBD41* transcript levels observed for line Y183F, despite high *AtPCO4 Y183F* transcription, likely arise due to the significantly lower activity of variant Y183F compared to variant C173A, although we cannot rule out that the introduction of mutations resulted in reduced *in vivo* stability of the PCO enzymes and therefore lower activity due to lower protein abundance. Nevertheless, overall the elevated HRG transcript levels in normoxia for lines C173A-1 and Y183F indicated that the variants were less active than *At*PCO4 WT *in vivo* and the phenotype of the *4pco At*PCO4 variant plants was more similar to *4pco AtPCO4* WT plants (Fig. S17*C*) than *4pco* plants (27), implying that the introduced *At*PCO4 variants were at least partially active *in vivo* in normoxia.

To assess if the plants expressing C173A and Y183F variants showed improved submergence tolerance compared to plants expressing *At*PCO4 WT, 10 plants of each line were submerged in the dark for 2.5 or 3.5 days alongside control Col-0 plants and dark-only (not submerged) controls. The plants were grown on soil and submerged fully in the dark to mimic flooding events of a field (murky water), to reduce secondary effects such as interference of light and therefore photosynthesis on our results, and to allow comparison with other similar studies (7, 9, 11, 43, 44). To minimize the effect of different survival and recovery due to plant age (45), plants with a phenotype equivalent to 3-week-old Col-0 were used for the submergence experiment. Survival and recovery rates after submergence were evaluated over a 10-days recovery period (Fig. 5, *C*–*F*). The results of the 2.5 day submergence experiment showed that more plants containing either *At*PCO4 C173A or Y183F survived and recovered than their Col-0 control plants (Fig. 5, *C* and *D*, Table S9, Fig. S20, left panel). In contrast, fewer plants of wt-1 and wt-2 survived and recovered compared to their Col-0 control plants (Fig. 5, *C* and *D*, Table S9). The fresh weight of 2.5-day submerged plants after 10 days of recovery was similar for variant lines, C173A-1, C173A-2 and Y183F, with plants from line Y183F being significantly heavier than wt-1 and wt-2 plants (Fig. S21, *A* and *B*, for dry weight see Fig. S21, *C* and *D*). However, the Col-0 control plants for line Y183F were also heavier than other Col-0 plants (Fig. S21, *A* and *B*). The differences in fresh weight were more substantial for plants recovering from 3.5 days submergence (Fig. S21, *E*–*H*). The fresh weight of recovered plants from lines, C173A-2 and Y183F, was significantly higher than the other lines, especially for plants from line Y183F (Fig. S21*F*). Nevertheless, the occurrence of zero survival of wt-1 + Col-0 and C173A-1 + Col-0 in at least one of the two experimental repeats (Fig. S20, right panel), affected this statistic as well as the reduced survival rate for both lines, wt-1 and C173A-1 (Fig. 5*E*). While the survival and recovery rates of wt-1 and C173A-1 were similar to their respective Col-0 plants, lines C173A-2 and Y183F appeared to survive and recover better after 3.5 days submergence than their Col-0 controls (Figs. 5, *E*, *F* and S20, right panel). Interestingly, a greater number of plants from line wt-2 survived (compared to its Col-0 control) after 3.5 days submergence (Fig. 5*E*), however, the surviving plants did not seem to grow and gain as much biomass as plants with *At*PCO4 Y183F (Figs. S21*F* and S20, right panel). A possible explanation could be that the lack of *At*PCO1, two and five in complemented *4pco* plants resulted in a prolonged stabilization of ERF-VIIs despite the re-introduced *At*PCO4 WT enzyme compared to Col-0. However, this stabilization seemed to be insufficient to promote biomass gain to the same extent as *At*PCO4 Y183F (Fig. S21*F*).

In summary, the potential for increasing HRG transcript levels in *4pco AtPCO4* variants lines C173A-1, -2 and Y183F, compared to wt-1 and wt-2, correlated with the reduced activity of the *At*PCO4 variants observed *in vitro*. The capability to increase stabilization of ERF-VIIs and thereby transcription of HRGs appeared to be beneficial for submergence tolerance, however, the results also demonstrated the importance of minimizing HRG transcription *via* ERF-VII destabilization during normoxia (line C173A-1). There are many potential confounding factors which make it challenging to directly ascribe improved submergence resilience with the altered catalytic features observed for the variants *in vitro*. Nevertheless, plants which were expressing *At*PCO4 variants with reduced biochemical activity showed improved recovery and survival of complemented *4pco* Arabidopsis plants subjected to submergence, especially when HRGs were not induced in normoxic conditions.

## Discussion

PCOs are critical oxygen-sensing enzymes that catalyze the first step in destabilizing ERF-VII transcription factors (11, 12). Their reduced activity at low O_2_ concentrations stabilizes ERF-VIIs to upregulate genes that enable acclimation to hypoxic conditions (7, 8, 11). This is important for plants to survive temporary periods of submergence. As climate change causes an increase in the intensity and duration of flood events, it is important for food security that crop plants are sufficiently resilient to this stress (46). Elevated stabilization of ERF-VIIs has been associated with improved submergence resilience (7, 21, 22).

The aim of our study was to identify residues that have a role in *At*PCO4 activity and substrate binding and to use this knowledge to engineer an *At*PCO4 variant with reduced activity that could increase submergence resilience in an Arabidopsis model. We demonstrated for the first time that slowing the activity of PCOs *via* single amino acid substitution at carefully selected residues has the potential to improve submergence resilience. Specifically, we reduced *At*PCO4 activity *in vitro* by mutating residues localized around the active site using a structure/function-guided approach. Although fluctuation in CD signal intensity suggests that we cannot rule out some degree of protein denaturation and thus a reduction in enzyme concentration (Figs. S7 and S8), overall the results indicate that the mutations affected iron binding, substrate binding and enzymatic catalysis. Kinetic and biophysical analysis of variants at selected residues confirmed their predicted contribution to ERF-VII Nt-Cys oxidation. *At*PCO4 variants of iron-coordinating residues, H98D and H100D, showed ablated activity, likely due at least in part to distorted iron binding capacity. *At*PCO4 Y73R also showed ablated activity, though this was due to severely weakened substrate binding. The more structurally conservative *At*PCO4 Y183F variant at the substrate binding site also caused weakened substrate affinity and consequently reduced (but not ablated) activity which is consistent with the data from the equivalent mutation in *Hs*ADO, Y212F (34). This residue is at the edge of a hairpin loop that is involved in inducing a conformational change upon substrate binding that primes *At*PCO4 for increased activity (47). Although residue Y183 may not be essential for priming, mutation to Phe could impact the effectiveness of this priming phenomenon. Modelled structures in which substrates are docked to the *At*PCO4 structure also suggest Y183 is important in stabilizing the active site (Figs. S4 and S5). Another possibility is that Y183 could interact with the Nt-Cys thiol as suggested for ADO (34, 37). Together, these data suggest that residue Y183 is important for substrate binding and potentially coordination of the substrate towards the active site iron rather than wider effects on *At*PCO4 structural changes.

Variants of residues closer to the catalytic site of *At*PCO4, D177E, V105G and S107L, reduced catalytic rate and efficiency but had no substantial impact on substrate affinity. The severely reduced activity of variant D177E might be due to the larger residue’s capability to mis-position the iron center, Nt-Cys, O_2_ or reaction intermediates, potentially linked to a rearrangement of secondary structure observed *via* CD (Fig. S7). Variant S107L likely showed lower activity due to reduced iron binding capacity, although this mutation could also have disturbed the positioning of O_2_, reaction intermediates and/or the *At*PCO4 D177-carboxylate group. Residue V105 might be involved in optimizing the position of the O_2_ co-substrate in the active site by limiting space elsewhere, an effect which was removed in the V105G variant to result in decreased catalytic efficiency. Variants of residues located *trans* to the substrate entry site, C173A and I175F, are further away from the Fe center so might have reduced the catalytic efficiency of *At*PCO4 by altering the position, and therefore the stability of Fe-oxygen/reaction intermediates. In a recent crystal structure of the homologous enzyme *Hs*ADO with a bound cyclic-peptide inhibitor coordinated towards the active site metal (PDB: 9DXB), the two residues equivalent to *At*PCO4 C173 (*Hs*ADO A202) and I175 (*Hs*ADO F204) are also distant from the Fe center implying that the relative position of these residues may remain similarly distant when the active site is occupied with substrate. The reduced catalytic efficiency of variant C173A with respect to O_2_ suggests that this mutation decreases the enzyme’s catalytic rate once O_2_ is coordinated to the active site, though whether this effect is through direct interaction with an intermediate or conformational change remains to be elucidated. Overall, our biochemical data were consistent with similar studies from other thiol dioxygenases; a more detailed discussion of the kinetic properties and how they may arise from potential structural impacts of the mutations at the *At*PCO4 active site can be found in the Supplementary Information file.

We selected two mutations that reduced the activity of *At*PCO4 *in vitro* to complement a *4pco* Arabidopsis plant model to investigate whether a decrease in *At*PCO4 activity could improve submergence tolerance. We selected one variant with a severe effect on *in vitro At*PCO4 activity, Y183F, which caused a reduction in substrate affinity, and one variant with a relatively mild effect on *At*PCO4 activity, C173A, which caused a reduction in catalytic turnover. We could generate two transgenic lines containing *AtPCO4 C173A* but could only obtain one line with *AtPCO4 Y183F*. A reason for this might be the low activity of *At*PCO4 Y183F which might stabilize *At*PCO4 substrates at unfavorably high levels *in vivo* under normal conditions and thereby reduce the germination/viability of seeds containing the *AtPCO4 Y183F* mutation. This hypothesis is supported by the low germination rate of the *4pco AtPCO4 Y183F* line that we obtained and its high *AtPCO4* transcript levels (Figs. S17–S19). It is feasible that, to complement *4pco* plants, a high level of *At*PCO4 Y183F expression was required to compensate for low *At*PCO4 Y183F activity. While we could obtain two separate lines with *AtPCO4 C173A*, their phenotypes showed some differences depending on *AtPCO4* transcript levels; line C173A-1 had similar *AtPCO4* transcript levels to lines Col-0, wt-1 and wt-2 and demonstrated some developmental delays and high HRG transcript levels under normal conditions, whereas line C173A-2 had increased *AtPCO4* transcript levels and showed growth patterns and HRG transcript levels similar to Col-0 and *4pco AtPCO4* WT plants (Figs. S17–S19). These results confirmed that mutation C173A reduces *At*PCO4 activity *in vivo* but also that enzymatic activity and expression levels together inform the plant phenotype.

In our experiment, both variants resulted in improved recovery after submergence following 2.5 and particularly 3.5 days of dark submergence. Reduced *At*PCO4 activity correlated with a ∼130 to 300% increase in recovery and ∼100 to 300% increase in survival probability compared to ∼60 to 200% and ∼50 to 280% for *At*PCO4 WT, respectively (both in relation to Col-0). While it is hard to dissect at which point during the submergence regime the decreased *At*PCO4 activity conferred the greatest advantage, the improved recovery implied that reduced *At*PCO4 activity was particularly beneficial during/after de-submergence. This is consistent with ERF-VII stability on reoxygenation contributing to post-hypoxic oxidative stress survival (48). *At*PCO4 transcript levels impacted normoxic HRG levels but may also have affected submergence tolerance, indicating that both the absolute amount of enzyme as well as its intrinsic activity are important in determining ERF-VII stability and HRG transcription. Notably in this study, reduced *At*PCO4 activity increased submergence tolerance to a greater degree when HRGs were not upregulated during normoxia, as was the case for C173A-2 compared to C173A-1, in accordance with previously published results that temporary but not constitutively stabilized ERF-VIIs are able to increase submergence tolerance (7, 49). When ERF-VIIs are stabilized during normoxia they promote fermentative metabolism, *e.g.*, by inducing the transcription of *ADH1* and *PDC1*, which consumes carbohydrate resources, thus, fewer resources will be available for the plant during submergence leading to lower submergence tolerance (7, 9, 49). It is feasible that this additional stress occurred in line C173A-1 and resulted in reduced survival and recovery compared to line C173A-2. Despite the potentially reduced resources before submergence, the submergence tolerance of C173A-1 was still improved after 2.5 days submergence compared to lines with *At*PCO4 WT, highlighting the fine-balance and importance of stabilized ERF-VIIs for submergence resilience.

Overall, our results demonstrate that we can engineer an *At*PCO4 enzyme with reduced but not ablated activity *in vitro* and can locate the biochemical cause. When we introduced the mutation into an Arabidopsis model, the resultant plant phenotype displayed increased submergence resilience. Although it is not possible to directly ascribe the improved stress response to the altered biochemical activity, due to an inability to control for factors *in planta* including stability of the expressed variant protein and susceptibility of the variant enzymes to cellular stress factors, we propose that the correlation between *At*PCO4 activity and biological phenotype supports our hypothesis that engineering PCO function can improve submergence resilience. The key residues and regions identified in our study are a suitable starting point for further PCO engineering efforts to achieve even more finely-tuned activities; balancing stabilization of ERF-VIIs at the onset/offset of hypoxia and destabilization during normoxia to avoid impacting yield.

Our experiments indicate that both absolute transcription levels of the introduced transgene and the catalytic ability of the expressed mutant PCOs are important in conferring improved submergence tolerance. Introducing targeted mutations *via* gene editing of genomic PCO sequences may in future negate the requirement to control for PCO transcript levels. Given the conservation of PCOs amongst flowering plants, this could also provide a route to engineer PCO activity and submergence resilience in flood-susceptible crop species.

## Experimental procedures

### Site-directed mutagenesis and protein production

The thrombin cleavage site in pET28a(+)-His-thrombin-AtPCO4-1 from White *et al.* 2018 was exchanged to a TEV cleavage site by Genscript using the *NcoI* and *XhoI* restriction sites. Specific sites in pET-28a(+)-His-TEV-AtPCO4-1 (recombinant protein synthesis, deposited at addgene #191802) and pENTR_PCO4_AtPCO4 were mutated using the QuikChange Site-Directed Mutagenesis Kit (Stratagene) protocol. Primers were designed according to Zheng, Baumann and Reymond (50) and are listed in Supplementary Table S1. After confirming successful mutation by sequencing, proteins from pET28a(+) plasmids were overexpressed in chemically competent *Escherichia coli* (*E. coli*) BL21 (DE3) cells and purified as described previously (17, 29) with some adaptations. In short, the filtered supernatant was purified using Ni(II)-affinity chromatography. The *At*PCO4 proteins were eluted during an imidazole concentration from 30 to 100%. After removal of imidazole using a desalting PD10 column, the His-tag was cleaved by incubating the proteins with TEV protease (purified from plasmid pRK793 (51) (addgene)) for 14 to 16 h. Cleaved protein was separated from uncleaved protein, His-tags and TEV protease *via* reverse Ni(II)-affinity chromatography. The untagged *At*PCO4 proteins were purified further by size exclusion chromatography and aliquots were stored at −80 °C. Protein concentration was determined by UV/Vis-spectroscopy and protein purity was estimated by SDS-PAGE.

### Determination of iron occupancy in *At*PCO4 enzymes

The iron binding capacity of *At*PCO4 variants was established using a colorimetric method with 1,10-phenanthroline and/or inductively coupled plasma mass spectrometry (ICP-MS) as described previously (17, 28). In short, to estimate the iron content using 1,10-phenanthroline, the protein samples were denaturated and the isolated iron was exposed to 1,10-phenathroline. The absorption of the Fe(II)-1,10-phenanthroline complex was measured at 535 nm and the Fe(II) content of each sample was calculated from a standard curve (0–250 μM FeSO_4_).

To determine the iron content using ICP-MS, the protein samples were diluted in 2% nitric acid and submitted to an ICP-MS autosampler. The instrument was calibrated with dilutions from two standards. The quality of the detection methods was tested with an external standard. The iron concentration of each sample was measured alongside an internal Rhodium standard to account for any instrumental drift. The mean of both iron concentrations measured was used to calculate the iron occupancy of the protein from the total protein concentration used to generate the sample. Statistical significance (*p* < 0.05) was determined in GraphPad Prism, Version 10.1.2.

### *At*PCO4 activity assay

Initial activity screens, optimization of exogenous iron concentrations, kinetic assays and O_2_ sensitivity assays were conducted, and the dioxygenation of the substrates was determined by MS as described previously (29). In short, enzyme master mixes (MM) were prepared in 50 mM bis-tris propane (1,3-bis[tris(hydroxymethyl)methylamino]propane) and 50 mM NaCl in LC/MS grade water and supplemented with FeSO_4_ and (+)-sodium l-ascorbate. Substrate MMs were prepared by resuspending RAP2.12_2-15_ (14-mer) peptide substrate in the same buffer as described above which was supplemented with tris(2-carboxyethyl)phosphine hydrochloride (TCEP) to prevent dimerization. Enzyme and substrate were pre-incubated for 5 to 10 min at 25 °C, before starting the reaction by adding the enzyme MM into the substrate MM. The reaction solution was quenched in 5% (v/v) formic acid at the respective time points and once an assay was finished, it was stored at - 20 °C. The samples were analyzed by high-throughput MS using a RapidFire RF360 sampling robot (Agilent) coupled to Agilent 6530 Accurate-Mass Q-TOF mass spectrometer operated in positive ion/electrospray mode. The source conditions were optimized for minimal fragmentation and maximal sensitivity. Samples were injected into a C4 solid phase extraction (SPE) cartridge, washed with equilibration solution (0.1% formic acid (v/v) in deionized water) and eluted in 85% acetonitrile with 0.1% formic acid (v/v). Mass spectra were analyzed in MassHunter Qualitative Analysis B.07.00 (Agilent) and areas under the chromatogram peaks of the substrate and oxidized product (+32 Da) were integrated using the RapidFire Integrator software (Agilent). Product concentrations were calculated by determining the proportion of the product from the total peptide concentration in the sample.

For the O_2_ sensitivity assay, the same principle was applied although using a different setup as previously described in detail (29).

### Binding assay using intrinsic tryptophan fluorescence

The affinity of RAP2.12_2-15_ peptide substrates to *At*PCO4 WT and variants was determined using the intrinsic fluorescence of a Trp residue close to the active site (W121 in *At*PCO4 isoform 2) as described previously (39). In short, iron was removed from *At*PCO4 enzymes to prevent substrate turnover by adding a dialysis step with 10 mM 1,10-phenanthroline and 50 mM ethylenediaminetetraacetic acid (EDTA) after the SEC step during protein purification. Samples were prepared under anaerobic conditions at room temperature. The iron removed enzymes were diluted in anaerobic assay buffer (50 mM Tris, 400 mM NaCl, pH 8) and supplemented with NiCl_2_. RAP2.12_2-15_ peptide substrate was dissolved in assay buffer, supplemented with TCEP, and diluted to the appropriate substrate concentrations. Enzyme and substrate solutions were dispensed into a 96-well black flat-bottom microplate in anaerobic conditions. The intrinsic Trp fluorescence of the enzyme was measured at λ_ex_/λ_em_ 280/350 nm, atmospheric conditions and 25 °C with gain adjustment (531) and a dynamic adjustment range of 530 to 600 nm using an optic filter module (FI 280 350, 1304A1) in a BMG PHERAstar microplate reader. Subsequently, the substrate and enzyme solutions were mixed and incubated for 10 min before measuring the intrinsic Trp fluorescence again as described above. Enzyme-substrate (ES) complex formation was determined by subtracting the first enzyme-only measurement from the measurement of the ES mixture and calculating the percentage of the quenching (change in intrinsic tryptophan fluorescence intensity (TFI)). Influences on fluorescence quenching not related to substrate binding were accounted for by using enzyme only controls for correction. The *K*_*d*_ was estimated by plotting the corrected TFI values over the respective substrate concentration and computing a non-linear regression function through the data points.

### *In silico* enzyme-substrate docking using HADDOCK2.4

Before uploading protein structures as PDB file to HADDOCK2.4, multiple occupancy side chains and water molecules were removed using the pdb_selaltloc PDB tool from https://wenmr.science.uu.nl/pdbtools/and PyMOL Molecular Graphics System (Version 3.0 Schrödinger, LLC) respectively. The charge state of iron in PDB 6S7E and nickel in 7REI was adjusted to Fe^2+^ and Ni^2+^. The structures of enzyme substrates RAP2.12 and RGS4 were predicted using AlphaFold2 and truncated to 5- and 7-mer peptides (RAP2.12_2-6/8_ and RGS5_2-6_) using PyMOL. The Nt-Cys of the peptide substrate was renamed to CYF to ensure the sulfur atom of the Nt-Cys side chain can coordinate to the iron centre of the enzyme. Modified PDB files from enzymes and peptide substrates were uploaded to HADDOCK2.4. For the enzyme, residues D177 and Y183 (in *At*PCO4, D206 and Y212 in *Hs*ADO) and the metal co-factor were selected as active residues and “Remove buried active/passive residues from selection” was de-selected. For the peptide substrates, residue two was selected as active residue. The N-terminal amino group of the peptide was modified to -NH_3_^+^. The default option was used for all other settings. Similar models of enzyme-substrate complexes were clustered together and their standard deviation from the average cluster was indicated by a z-score. Clusters closest to the average cluster were chosen for final visualization in PyMOL.

### *At*PCO4 *in planta* experiments

#### Growth conditions

Col-0 was used as wildtype ecotype (seeds obtained from Dept. of Biology, University of Oxford). Plants were grown with a 16 h photoperiod at 22 °C in a controlled environment room. At the end of a plant’s life cycle, it was transferred into a drying room until seed harvest. Plants used for the submergence experiment and RT-qPCR analysis were grown in heavier compost consisting of 1:1 modular seed and John Innes No.2 with one-fourth fine vermiculite.

#### Preparation of plasmids for transformation into Arabidopsis plants

The mutations, C173A and Y183F, and the insertion of E135 to modify *At*PCO4-1 to *At*PCO4-2 were introduced into the entry vector pENTR223:PCO4 vector (Arabidopsis biological resource centre (ABRC) stock code G82286) that encodes *At*PCO4-1 using site-directed mutagenesis (described above, Table S1). After successful mutagenesis and amplification of the plasmids, the *AtPCO4-2* mutants were transferred into the destination vector pH7WG-promPCO4 (previously described in White *et al.* 2020) using Gateway Cloning, in particular, LR recombination. In more detail, entry and destination vector were incubated with LR clonase II in 1 x TE buffer (10 mM Tris-Cl, 1 mM EDTA, pH 8.0) for 16 to 18 h at 25 °C. Proteinase K was added and the solution was incubated for 10 min at 37 °C to stop the reaction. The recombination solution was transformed into ccdB susceptible *E. coli* DH5α cells. Successfully recombined plasmids, *i.e.*, pHWG-promPCO4-*At*PCO4-2-C173A and -Y183F, were confirmed by sequencing with primer sets attB1+2 and PCO4_F+R (Table S2) and transformed into the *Agrobacterium* strain GV3101. Successful colonies were confirmed by PCR using primer sets as described above.

#### Transformation of *Arabidopsis* plants

To create *4pco AtPCO4-2* WT and mutant plants, the floral dip method (52) was used on the previously described triple knockout Arabidopsis plants (27) in Col-0 background, *3pco+AtPCO5+/−* (*AtPCO1−/−, AtPCO2−/−, AtPCO4−/−* and *AtPCO5+/−*). This line had been used previously to generate *4pco* plants as *4pco* plants cannot self-fertilize (18, 27) and is based on T-DNA mutant lines from the Nottingham Arabidopsis Stock Centre (uNASC) as follows T-DNA insertion line N451210 for *At*PCO1 (At5g15120), N116554 for *At*PCO2 (At5g39890), N471015 for *At*PCO4 (At2g42670) and N684949 for *At*PCO5 (At3g58670) (27). For the transformation, agrobacteria colonies containing the respective recombined pHWG-promPCO4-AtPCO4-2 plasmid were cultured in LB medium supplemented with 20 ng mL^–1^ rifampicin, 50 ng mL^–1^ gentamycin, 100 ng mL^–1^ spectinomycin, and 300 ng mL^–1^ streptomycin, at 28 °C and 140 to 160 rpm until the suspension turned turbid (∼18–24 h). The cells were pelleted and resuspended in 5% sucrose supplemented with Silwett L-77. The inflorescences of *3pco+AtPCO5+/−* Arabidopsis plants were dipped into the agrobacterium-sucrose suspension and stored in the dark for 12 h. This procedure was repeated after 7 days for a higher success rate. Once the growth cycle was finished, the seeds were harvested and dried.

#### Selection of 4pco AtPCO4-2 lines

After stratification of the seeds for 7 to 10 days, the seeds were spread on Hygromycin selection plates. Promising seedlings were transferred onto soil (3:1 modular seed compost and fine vermiculite). After 2 to 3 weeks the plants were genotyped for *AtPCO4* insertion as well as for *AtPCO5* WT and *AtPCO5* T-DNA using a piece of their leaf tissue (for primer list see Table S3) to determine *4pco AtPCO4-2* WT and mutant lines. The tissue was ground in DNA extraction buffer (200 mM Tris, 250 mM NaCl, 25 mM EDTA, 0.5% SDS, pH 7.5) and the suspension was centrifuged at maximal speed (∼15,000*g*) for 2 min. The DNA was precipitated from the supernatant by adding an equal amount of isopropanol and incubating the sample for 5 min. The DNA was pelleted at maximum speed (∼15,000*g*) for 5 min, the supernatant was discarded, and the pellet was dried. After resuspending the DNA in sterile ddH_2_O, the DNA was added to the PCR mix containing GoTaq mix, the appropriate primer pairs and DMSO. The PCR program included a 2 min denaturation at 95 °C, before 30 cycles of denaturation at 95 °C for 30 s, annealing at 5 °C below the melting temperature (T_m_) of the primers for 30 s and elongation at 72 °C for 1 min. The final step was a final elongation for 10 min at 72 °C before storing the PCR products at 4 °C until separating them on a 1% agarose gel. In addition, the sequence of the inserted *AtPCO4-2* was confirmed by sequencing (using primer pairs listed in Tables S2 and S3).

### RT-qPCR

RT-qPCR analysis was performed on copy DNA (cDNA) from 5.5-week-old plants. RNA was extracted from four plants per line using a Plant RNA purification kit. RNA concentrations were measured at an absorbance of 260 nm and calculated using a UV/Vis spectrophometer. The mRNA was transcribed into cDNA using a cDNA synthesis kit. The cDNA was mixed with the qPCRBIO SyGreen Mix Hi-ROX. Appropriate primer pairs (Table S4) were added and the reactions were run on a real-time PCR system. The cycle threshold (C_T_) of each target was normalized to the C_T_ of the housekeeping gene, UBQ10, to determine relative quantities of the transcript levels (ΔC_T_). The fold change (ΔΔC_T_) between mRNA present in *4pco AtPCO4-2* lines compared to Col-0 was calculated by normalizing the ΔC_T_ from the *4pco AtPCO4-2* lines to ΔC_T_ of Col-0.

### Submergence experiment

Plants were submerged in 26 cm tap water in black boxes that were additionally wrapped in aluminum foil to ensure complete darkness. Each box contained 10 × *4pco AtPCO4-2* WT or mutant plants and 10 × Col-0 plants as control. All plants were submerged at the 9-leaf stage at the end of a light cycle (after ∼15 h of light). Dark controls were grown under aerobic conditions in the dark for the maximal submergence period. After de-submergence, the plants were covered with a transparent lid for 24 h to enable recovery in humid conditions. A yes/no system was applied to score survival and recovery rates during the 10 days after submergence. On day 10 of recovery, the fresh weight of the rosettes was determined. The dry weight was measured after drying the rosettes at 50 °C until no weight difference between days could be observed.

## Data availability

All data described are available in this manuscript and in the Supporting Information.

## Supporting information

The Supplemental Information for this article contains Tables S1 – S9, Figures S1 – S20, and a Supplementary Discussion (7, 15, 16, 18, 26, 27, 32, 33, 34, 38, 39, 40, 41, 42, 53).

## Conflict of interests

The authors declare that they have no conflicts of interest with the contents of this article.
