# Supplementary material for: Rational engineering of the Arabidopsis thaliana plant cysteine oxidase 4 active site can reduce biochemical activity and improve submergence tolerance
Source: J Biol Chem. 2026 Jul 13;302(9):113331. doi: 10.1016/j.jbc.2026.113331 (PMC13485640; doi:10.1016/j.jbc.2026.113331)
Supplement: Supplementary file 1 [file mmc1.docx]

**SUPPLEMENTARY INFORMATION**

Rational engineering of the *Arabidopsis thaliana* plant cysteine oxidase 4 active site can reduce biochemical activity and improve submergence tolerance

Summary title: Engineered *At*PCO4 active site effects *in vitro* and *in vivo*

**Authors/affiliations:**

Anna Dirr^1^, Arianna Del Greco^2,3^, Simon Howe^2^, Leah S. Walker^2^, Monica Perri^2^, Vinay Shukla^2^, Francesco Licausi^2^, Emily Flashman^2*^

^1^Department of Chemistry, University of Oxford, United Kingdom

^2^Department of Biology, University of Oxford, United Kingdom

^3^University of Pisa, Biology Department, Via L. Ghini 13 - 56126, Pisa, Italy

*Corresponding author: Emily Flashman, emily.flashman@biology.ox.ac.uk

**Table of contents**

**Supplementary Tables**

[Table S1. Primer sequences for *AtPCO4* mutagenesis.](#_Toc216252817)

[Table S2. Primers for confirming complementation of *4pco* plants with *AtPCO4* WT and mutants.](#_Toc216252818)

[Table S3. Primer pairs for genotyping.](#_Toc216252819)

[Table S4. RT-qPCR primer sequences.](#_Toc216252820)

[Table S5. Michaelis-Menten kinetics of *At*PCO4 enzymes using 0 – 1000 μM RAP.12_2–15_.](#_Toc216252821)

[Table S6. Leaf perimeter.](#_Toc216252822)

[Table S7. Canopy size.](#_Toc216252823)

[Table S8. Leaf number.](#_Toc216252824)

[Table S9. Survival and recovery probability normalised to respective Col-0.](#_Toc216252825)

**Supplementary Figures**

[Figure S1. PCOs as oxygen sensors in the Cys/Arg-N-degron pathway.](#_Toc216252869)

[Figure S2. Structural difference between *At*PCO4-1 and -2 and their *in vitro* activities.](#_Toc216252870)

[Figure S3. Iron occupancy, activity and substrate binding models of His-triad variants H98D and H100D.](#_Toc216252871)

[Figure S4. Substrate binding of *Hs*ADO, *At*PCO4-2 and *Hs*CDO-1.](#_Toc216252872)

[Figure S5. *In silico* docking models of ERF-VII and non-ERF-VII substrates to *At*PCO4-2 WT using Haddock2.1.](#_Toc216252873)

[Figure S6. Overview of recombinant purified *At*PCO4 WT and variants.](#_Toc216252874)

[Figure S7. CD spectra of active site variants.](#_Toc216252875)

[Figure S8. CD spectra of iron removed AtPCO4 enzymes supplemented with nickel.](#_Toc216252876)

Figure S9. Optimisation of supplemental iron for AtPCO4-2 wt and variants.

[Figure S10. Iron occupancy and O_2_ sensitivity of variants with mutations at the substrate entry site.](#_Toc216252877)

[Figure S11. Substrate binding and iron occupancy of variants with mutations *trans* to His-triad.](#_Toc216252878)

[Figure S12. Substrate binding and iron occupancy of variants with mutations *trans* to the substrate entry site.](#_Toc216252879)

[Figure S13. Complementation of *4pco* Arabidopsis plants with *AtPCO4* *WT*, *C173A* and *Y183F*.](#_Toc216252880)

[Figure S14. Sequencing results from complemented lines wt-1 and -2*.*](#_Toc216252881)

[Figure S15. Sequencing results from complemented lines C173A-1 and -2*.*](#_Toc216252882)

[Figure S16. Sequencing results from complemented line Y183F*.*](#_Toc216252883)

[Figure S17. Phenotype of *4pco AtPCO4* complemented lines.](#_Toc216252884)

[Figure S18. *AtPCO4* and *5* transcript levels of 5.5-week-old *4pco AtPCO4* plants.](#_Toc216252885)

[Figure S19. Statistical significance of differences in HRG transcript levels of *4pco AtPCO4* plants.](#_Toc216252886)

[Figure S20. Submergence recovery of all *4pco* lines and their Col-0 control plants.](#_Toc216252887)

[Figure S21. Fresh and dry weight of *4pco AtPCO4* *WT*, *C173A*, *Y183F* and Col-0 control plants after 2.5 and 3.5 d submergence.](#_Toc216252888)

**Supplementary Discussion**

Additional discussion of the kinetic properties and predicted structural properties of *At*PCO4 variants

- Fe(II)-binding Residues: H98 and H100
- Substrate-binding Residues: Y73 and Y183
- Residues trans to the His-triad: Asp177, Val105 and Ser107
- Residues trans to the substrate entry site: C173 and I175

**Supplementary References**

# Supplementary Tables

Table S1. Primer sequences for *AtPCO4* mutagenesis.

Primer names specify the encoded mutation and the name of a restriction enzyme which recognises an incorporated silent mutation.

| **mutation** | **5' – 3'** |
| --- | --- |
| AtPCO4-1_Q134_A135insE_F | GATCCATCACAAGAAGCAAGACCTGCTAAACTGGTGAAGGATAC |
| AtPCO4-1_Q134_A135insE_R | GTTTAGCAGGTCTTGCTTCTTGTGATGGATCCTCTGGTTCG |
| **active site mutation** |  |
| Y73R_XmiI_F | GCAATAAAGCGTCTACATTTGCATGAGTGTGACAGTTTC |
| Y73R_XmiI_R | GCAAATGTAGACGCTTTATTGCTGGGGGAGACTGATTAC |
| H98D_MamI_F | GATACCTCTTGATAACCATCCGGGCATGACCGTGCTAAG |
| H98D_MamI_R | GATGGTTATCAAGAGGTATCATAGAAGAAGGTGGCATACAGAAG |
| H100D_SmaI_F | CTTCATAACGACCCGGGCATGACCGTGCTAAG |
| H100D_SmaI_R | CATGCCCGGGTCGTTATGAAGAGGTATCATAG |
| V105G_F | CATGACCGGTCTAAGCAAGCTCGTTTATGGTTCAATG |
| V105G_R | CTTGCTTAGACCGGTCATGCCCGGATGGTTATGAAG |
| S107L_AflII_F | CATGACCGTGCTACTTAAGCTCGTTTATGGTTCAATGCATGTG |
| S107L_AflII_R | CATAAACGAGCTTAAGTAGCACGGTCATGCCCGGATGGTTATG |
| C173A_SphI_F | CAAAGCCATCACGCATGCGGCTATTCTTGACATCTTAGCTCCAC |
| C173A_SphI_R | CAAGAATAGCCGCATGCGTGATGGCTTTGAAACAGTGAATGTTG |
| I175F_NsbI_F | CATCACCCATTGCGCATTTCTTGACATCTTAGCTCCACCTTAC |
| I175F_NsbI_R | GTCAAGAAATGCGCAATGGGTGATGGCTTTGAAACAGTGAATG |
| D177E_BglII_F | CTATTCTTGAGATCTTAGCTCCACCTTACTCTTC |
| D177E_BglII_R | GCTAAGATCTCAAGAATAGCACAATGGGTGATG |
| Y183F_F(1) | ACATCTTAGCTCCACCTTTCTCTTCAGAGCATGATC |
| Y183F_R(1) | GATCATGCTCTGAAGAGAAAGGTGGAGCTAAGATGT |
| **reversion of spontaneous mutation in *AtPCO4-2* in pENTR_PCO4_AtPCO4-2** | |
| I148M_F | GATACTGAGATGACGGCTCAAAGCCCAGTAACAAC |
| I148M_R | CTTTGAGCCGTCATCTCAGTATCCTTCACCAGTTTAGC |

Table S2**.** Primers for confirming complementation of *4pco* plants with *AtPCO4* WT and mutants.

| **primer** | **5’ – 3’** | **comment** |
| --- | --- | --- |
| attB1  attB2 | ACAAGTTTGTACAAAAAAGCAGGCT  ACCACTTTGTACAAGAAAGCTGGGT | Gateway^®^ Technology, Invitrogen |
| PCO4_F  PCO4_R | GCCTTACTTTGCTCAGAGGCTTTAC  CAAGTTCTAATGACAGGACCTCTGTACG |  |
| pPCO4_F  pPCO4_R | CAGAATTCGCCCTTTTCACCGTTC  CACTAGTGATCTGAATCCAATTCAAAAG | covering promotor region of *AtPCO4* |

Table S3. Primer pairs for genotyping.

| **primer** | **5’ – 3’** |
| --- | --- |
| insPCO5_F(2)  insPCO5_R(2) | GCCCATTTAGGTAGCTGCAGTG  AGCTTCCTGTTCGAGACCAA |
| LBb1 (2) | GCGTGGACCGCTTGCTGCAACT |
| promotorPCO4_F | [ATGTGCGATTCATTTCCCAA](https://www.thermofisher.com/order/catalog/product/10336022?tsid=Email_POE_OC_OrderConfirm%20%20_SKULINK) |
| complPCO4_R | [GGGCTTTGAGCCGTCATCTCAGTATCCTTC](https://www.thermofisher.com/order/catalog/product/10336022?tsid=Email_POE_OC_OrderConfirm%20%20_SKULINK) |
| PCO4_F  PCO4_R | GCCTTACTTTGCTCAGAGGCTTTAC  CAAGTTCTAATGACAGGACCTCTGTACG |

Table S4. RT-qPCR primer sequences.

| **target** | **name** | **5’ – 3’** | **source** |
| --- | --- | --- | --- |
| **Ubiquitin10 (At4g05320) (UBQ10)** | AT4G05320_F | GGCCTTGTATAATCCCTGATGAATAAG | Licausi *et al*., 2011(3) |
|  | AT4G05320_R | AAAGAGATAACAGGAACGGAAACATAGT |  |
| ***AtPCO4***  **(At2g42670)** | PCO4_KO_1_F | GAACGCAATGGAAGTAATCAGTCTC | Dr Anna Dirr |
|  | PCO4_KO_1_R | TCATAGAAGAAGGTGGCATACAGAA |  |
|  | qpAtPCO4-2_F | ACCAGAGGATCCATCACAAGAAGC | Dr Anna Dirr |
|  | qpAtPCO4-2_R | TACTGGGCTTTGAGCCGTCATC |  |
|  | sgPCO4_F | CGAACCAGAGGATCCATCACAAGA | Prof Francesco Licausi |
|  | sgPCO4_R | GCCGTCATCTCAGTATCCTTCACC |  |
|  | PCO4_White_F | CATGAGTGTGACAGTTTCTCTATAG | White *et al.*, 2020(1) |
|  | PCO4_White_R | TGGTTCGGTCAGTTGAGGCTCTA |  |
| ***AtPCO5***  **(At3g58670)** | AtPCO5_1_F | CGACATCTTATCTCCTCCATACTCT | Dr Anna Dirr |
|  | AtPCO5_1_R | CATTCATCACTTCAATCTCACCAGG |  |
| ***ADH1***  **(At1g77120)** | ADH_F | TATTCGATGCAAAGCTGCTGTG | Licausi *et al*., 2011(3) |
|  | ADH_R | CGAACTTCGTGTTTCTGCGGT |  |
| ***PDC1***  **(At4g33070)** | PDC1_F2 | GGACACCAAAATCGGATCGAT C | Masson *et al.*, 2019(2) |
|  | PDC1_R2 | CTACTGAGGATTGGGAGGACG |  |
| ***LBD41***  **(At3g02550)** | LBD41_F | TGAAGCGCAAGCTAACGCA | Licausi *et al*., 2011(3) |
|  | LBD41_R | ATCCCAGGACGAAGGTGATTG |  |
| ***SAD6***  **(At1g43800)** | SAD6_F | TTGGCAACCCGCTTCTTTCTTACC | Masson *et al.,* 2019(2) |
|  | SAD6_R | TTTCCCTCAGCTCACGAACCTG |  |
| ***HB1***  **(At2g16060)** | HB1_F | ATGGAGAGTGAAGGAAAGATTGTG | Gibbs *et al.*, 2018(4) |
|  | HB1_R | TTAGTTGGAAAGATTCATTTCAGC |  |
| ***SUS4***  **(At3g43190)** | SUS4_F | AACGCAGAACGTGTAATAACG | Gibbs *et al.*, 2018(4) |
|  | SUS4_R | CTCGGAGTGATGTTGAGTCC |  |

Table S5. Michaelis-Menten kinetics of *At*PCO4 enzymes using 0 – 1000 μM RAP.12_2–15_.

Y183F values were determined from data for 0 – 2000 μM RAP.12_2–15_ (Supplementary Figure S9C). ± indicates the SEM.

| ***At*PCO4** | ***V_max_*** | | | ***K_M_*** | | | | | ***k_cat_*** | | ***k_cat_/K_M_*** | | |  |
| --- | --- | --- | --- | --- | --- | --- | --- | --- | --- | --- | --- | --- | --- | --- |
|  | μmol.mg^–1^.min^–1^ | | | | μM | | min^–1^ | | | | | min^–1^.μM^–1^ | | |
| **WT** | **2.52** | **±0.07** | **100.30** | | | **±9.90** | | **69.55** | | **±1.97** | **0.69** | | **±0.20** |  |
| **Y73R** | 0.04 | ±0.003 | 51.20 | | | ±12.28 | | 1.12 | | ±0.08 | 0.02 | | ±0.006 |  |
| **V105G** | 0.29 | ±0.01 | 97.44 | | | ±15.54 | | 7.94 | | ±0.35 | 0.08 | | ±0.02 |  |
| **S107L** | 0.49 | ±0.03 | 98.91 | | | ±18.60 | | 13.61 | | ±0.86 | 0.14 | | ±0.05 |  |
| **C173A** | 2.51 | ±0.13 | 398.80 | | | ±46.85 | | 69.20 | | ±3.48 | 0.17 | | ±0.07 |  |
| **I175F** | 1.81 | ±0.06 | 294.00 | | | ±23.32 | | 50.01 | | ±1.67 | 0.17 | | ±0.07 |  |
| **D177E** | 0.13 | ±0.01 | 267.00 | | | ±55.23 | | 3.53 | | ±0.32 | 0.01 | | ±0.006 |  |
| **Y183F** | 1.19 | ±0.09 | 1562.00 | | | ±206.40 | | 32.90 | | ±2.49 | 0.02 | | ±0.01 |  |

Table S6. Leaf perimeter.

Determined by analysing respective plant photos using Leaf-GP(5) and displayed in mm.

|  | **Col-0** | | | **wt-1** | | | | **wt-2** | | | **C173A-1** | | | **C173A-2** | | | **Y183F** | | | |
| --- | --- | --- | --- | --- | --- | --- | --- | --- | --- | --- | --- | --- | --- | --- | --- | --- | --- | --- | --- | --- |
| **Time (days)** | **mean** | **SD** | **n** | | **mean** | **SD** | **n** | **mean** | **SD** | **n** | **mean** | **SD** | **n** | **mean** | **SD** | **n** | **mean** | **SD** | **n** |  |
| **7** | 4.7 | 5.2 | 118 | | 3.4 | 0.4 | 19 | 3.1 | 0.4 | 6 |  |  | 0 | 3.4 | 0.5 | 15 | 2.4 | 0.1 | 2 |  |
| **9** | 4.1 | 1.1 | 42 | | 10.5 | 8.3 | 45 | 3.6 | 0.9 | 27 | 3.5 | 0.3 | 4 | 4.8 | 1.2 | 54 | 3.7 | 0.3 | 7 |  |
| **14** | 16.2 | 4.3 | 216 | | 16.7 | 3.6 | 48 | 12.1 | 3.3 | 48 | 10 | 3 | 47 | 17.2 | 4.2 | 72 | 11.3 | 4.7 | 19 |  |
| **15** | 17.3 | 4.5 | 48 | | 20.6 | 3.7 | 48 | 14.6 | 3.7 | 48 | 11.4 | 3.1 | 48 | 20.7 | 4.8 | 72 | 12.6 | 5.4 | 20 |  |
| **16** | 21.5 | 5.7 | 48 | | 25.7 | 5.1 | 48 | 17 | 4.4 | 48 | 13.3 | 3.2 | 48 | 26.1 | 5.6 | 72 | 14.4 | 6.7 | 21 |  |
| **17** | 26.6 | 6.4 | 48 | | 31.3 | 6.3 | 48 | 20.7 | 5.3 | 48 | 15.2 | 3.9 | 48 | 32.1 | 6.7 | 72 | 17.2 | 7.7 | 21 |  |
| **18** | 45.7 | 5.7 | 89 | | 39.9 | 6.6 | 47 | 25.4 | 5.6 | 48 | 18.1 | 4.3 | 48 | 42.4 | 7.8 | 64 | 22.9 | 9.7 | 21 |  |
| **19** | 36.8 | 7.4 | 49 | | 37.7 | 5.5 | 7 | 31.4 | 6.9 | 44 | 22.7 | 5.6 | 48 | 37.7 | 6.2 | 14 | 30.8 | 10.7 | 19 |  |
| **20** | 44.8 | 8.5 | 40 | | 44.5 | 6.3 | 7 | 38.3 | 8.5 | 44 | 26.6 | 6.1 | 48 | 45 | 6.5 | 14 | 33.6 | 13.2 | 12 |  |
| **21** | 50.2 | 14.5 | 70 | | 40.6 | 11.6 | 7 | 39.3 | 7.3 | 17 | 32.9 | 6.3 | 52 | 45.2 | 9.6 | 14 | 39.1 | 13.9 | 12 |  |

Table S7. Canopy size.

Determined by analysing respective plant photos using Leaf-GP(5) and displayed in mm^2^.

|  | **Col-0** | | | **wt-1** | | | | **wt-2** | | | **C173A-1** | | | **C173A-2** | | | **Y183F** | | | |
| --- | --- | --- | --- | --- | --- | --- | --- | --- | --- | --- | --- | --- | --- | --- | --- | --- | --- | --- | --- | --- |
| **Time (days)** | **mean** | **SD** | **n** | | **mean** | **SD** | **n** | **mean** | **SD** | **n** | **mean** | **SD** | **n** | **mean** | **SD** | **n** | **mean** | **SD** | **n** |  |
| **7** | 2.2 | 6.8 | 118 | | 0.6 | 0.1 | 19 | 0.5 | 0.1 | 6 |  |  | 0 | 0.7 | 0.1 | 15 | 0.4 | 0 | 2 |  |
| **9** | 1 | 0.4 | 42 | | 10.3 | 13.9 | 45 | 0.7 | 0.3 | 27 | 0.7 | 0.1 | 4 | 1.1 | 0.3 | 54 | 0.7 | 0 | 7 |  |
| **14** | 8.3 | 3.4 | 216 | | 8.5 | 2.6 | 48 | 4.8 | 2 | 48 | 3.5 | 1.7 | 47 | 9 | 3.3 | 72 | 4.5 | 2.6 | 19 |  |
| **15** | 9.2 | 3.9 | 48 | | 11.7 | 3.2 | 48 | 6.5 | 2.6 | 48 | 4.3 | 1.9 | 48 | 12.2 | 4.5 | 72 | 5.4 | 3.1 | 20 |  |
| **16** | 13.3 | 5.2 | 48 | | 16.8 | 4.8 | 48 | 8.8 | 3.7 | 48 | 5.8 | 2.3 | 48 | 17.1 | 5.4 | 72 | 7.1 | 4.5 | 21 |  |
| **17** | 18.5 | 6.6 | 48 | | 24 | 6.6 | 48 | 12.3 | 5.1 | 48 | 7.6 | 3 | 48 | 24.5 | 7.5 | 72 | 9.7 | 6 | 21 |  |
| **18** | 41.5 | 7 | 89 | | 34.4 | 8.3 | 47 | 16.8 | 6.1 | 48 | 10 | 3.7 | 48 | 35.7 | 9.9 | 64 | 14.8 | 8.6 | 21 |  |
| **19** | 30.1 | 8.6 | 49 | | 30.5 | 7.7 | 7 | 23.9 | 8.5 | 44 | 14.1 | 5.3 | 48 | 28.4 | 6.9 | 14 | 23.7 | 11.5 | 19 |  |
| **20** | 41.4 | 11.2 | 40 | | 38.5 | 9.2 | 7 | 32.3 | 11.5 | 44 | 19.6 | 8.7 | 48 | 37.8 | 8.5 | 14 | 27.8 | 14.8 | 12 |  |
| **21** | 53.1 | 19.6 | 70 | | 42.1 | 11.2 | 7 | 33.1 | 9.7 | 17 | 26.7 | 8.2 | 52 | 46.6 | 10.1 | 14 | 35.1 | 18.1 | 12 |  |

Table S8. Leaf number.

Determined by analysing respective plant photos using Leaf-GP(5).

|  | **Col-0** | | | **wt-1** | | | | **wt-2** | | | **C173A-1** | | | **C173A-2** | | | **Y183F** | | | |
| --- | --- | --- | --- | --- | --- | --- | --- | --- | --- | --- | --- | --- | --- | --- | --- | --- | --- | --- | --- | --- |
| **Time (days)** | **mean** | **SD** | **n** | | **mean** | **SD** | **n** | **mean** | **SD** | **n** | **mean** | **SD** | **n** | **mean** | **SD** | **n** | **mean** | **SD** | **n** |  |
| **7** | 2.6 | 1.9 | 118 | | 2 | 0 | 6 | 2 | 0 | 19 |  |  | 0 | 2.1 | 0.2 | 15 | 2.5 | 0.5 | 2 |  |
| **9** | 3.1 | 1.5 | 42 | | 2.7 | 1.4 | 27 | 6.4 | 4.7 | 45 | 2.3 | 0.4 | 4 | 3.5 | 1.2 | 54 | 2.7 | 0.5 | 7 |  |
| **14** | 7.1 | 1.9 | 216 | | 6.2 | 1.6 | 48 | 8 | 1.9 | 48 | 5.8 | 1.5 | 47 | 7.1 | 1.8 | 72 | 5.9 | 1.9 | 19 |  |
| **15** | 7.9 | 2.1 | 48 | | 6.8 | 1.9 | 48 | 8.4 | 1.9 | 48 | 6 | 1.6 | 48 | 8.2 | 1.9 | 72 | 6.4 | 2.5 | 20 |  |
| **16** | 8.5 | 2.3 | 48 | | 7.9 | 2.4 | 48 | 8.5 | 2 | 48 | 6.3 | 1.8 | 48 | 8.8 | 2.1 | 72 | 6.6 | 2.4 | 21 |  |
| **17** | 9.1 | 2.3 | 48 | | 8.4 | 2.2 | 48 | 9.3 | 1.8 | 48 | 7.6 | 2.1 | 48 | 9.1 | 2.2 | 72 | 6.6 | 2.2 | 21 |  |
| **18** | 10.9 | 2.6 | 89 | | 8.4 | 2 | 48 | 9.7 | 1.8 | 47 | 7.7 | 2.5 | 48 | 9.9 | 2.2 | 64 | 7.3 | 2.5 | 21 |  |
| **19** | 9.9 | 2.2 | 49 | | 9.1 | 2.3 | 44 | 9.6 | 1.2 | 7 | 8.8 | 2.4 | 48 | 8.9 | 1.8 | 14 | 8.4 | 2.3 | 19 |  |
| **20** | 11.3 | 2.1 | 40 | | 10.5 | 2.1 | 44 | 10.7 | 2.5 | 7 | 8.8 | 2.5 | 48 | 11.8 | 2.2 | 14 | 9.3 | 3.6 | 12 |  |
| **21** | 14.7 | 4.9 | 70 | | 9.9 | 2.1 | 17 | 17.1 | 3.8 | 7 | 9.3 | 2.7 | 52 | 17.4 | 2.8 | 14 | 10.4 | 1.9 | 12 |  |

Table S9. Survival and recovery probability normalised to respective Col-0.

| % compared to Col-0 | | | | | | |
| --- | --- | --- | --- | --- | --- | --- |
|  |  | **wt-1** | **wt-2** | **C173A-1** | **C173A-2** | **Y183F** |
| 2.5 days | **survival** | 50 | 83 | 145 | 300 | 125 |
|  | **recovery** | 80 | 57 | 300 | 157 | 128 |
| 3.5 days | **survival** | N/A | 275 | 100 | 233 | 118 |
|  | **recovery** | N/A | 200 | 200 | 280 | 152 |

# Supplementary Figures

Figure S1. PCOs as oxygen sensors in the Cys/Arg-N-degron pathway.

PCOs catalyse the dioxygenation of the N-terminal Cys of their substrates, mainly group VII ethylene response factors (ERF-VII, ERF in scheme), in normoxia (atmospheric O_2_) marking them for degradation. At low O_2_ levels (hypoxia) the activity of PCOs is reduced, wherefore ERF-VIIs are stabilised and induce the transcription of hypoxia responsive genes (HRGs). The cone on the left indicates reduction in O_2_ levels from top to bottom. ATE: arginyl-transferase, PRT6: proteolysis 6, 26 S: 26 S proteosome.

Figure S2. Structural difference between *At*PCO4-1 and -2 and their *in vitro* activities.

**(A)** Cartoon presentation of *At*PCO4-1 with indicated additional residue in isoform 2 (PDB: 6S7E). **(B)** Deconvoluted mass spectrum of the dioxygenated (+ 32 Da) RAP2.12_2-15_ peptide (1442.7 Da) catalysed by *At*PCO4-2 (10 min reaction time). **(C)** and **(D)** Activity comparison of *At*PCO4-1 and -2 with and without His-tag. Activities are shown as turnover of μmol substrate per mg enzyme. The assays were performed in technical triplicates (n = 3), values are mean ± standard deviation (as indicated by error bars). Assays comprised 0.4 μM enzyme from two purified batches of *At*PCO4-1 and one batch of *At*PCO4-2 at 25 °C in reaction buffer (50 mM bis-tris propane (pH 8), 50 mM NaCl) with 5 μM FeSO_4_, 1 mM l-ascorbate, 1 mM TCEP and 200 μM RAP2.12_2–15_. His-*At*PCO4-1 and -2 converted 63% and 43% substrate (~ turnover number of 157 and 109, respectively). *At*PCO4-1 and
-2 converted 69% and 76% substrate (~ turnover number of 172 and 190, respectively). The data showed that differences between the enzymes were not significant (ns) for P < 0.05 after performing one-way ANOVA with Holm-Šídák's test (GraphPad Prism, Version 10.1.2).

Figure S3. Iron occupancy, activity and substrate binding models of His-triad variants H98D and H100D.

**(A)** Cartoon representation of *At*PCO4 active site obtained from a crystal structure (PDB: 6S7E) with His-triad residues highlighted in red. **(B)** *At*PCO4 WT (technical replicates, n = 9) and variant (technical replicates, n = 3) activity with 200 μM RAP2.12_2-15_ in assay buffer (50 mM bis-tris-propane, pH 8, 50 mM NaCl) with 1 mM TCEP and 1 mM l-ascorbate. Statistical significance was determined using one-way ANOVA and Holm-Šídák's testing to correct for multiple comparisons (GraphPad Prism). **(C)** Iron occupancy determined using ICP-MS for WT and variant H100D (n = 2) and a colorimetric assay for variant H98D (technical replicates, n = 3). Statistical significance of all active site variants compared to WT was determined using one-way ANOVA and Holm-Šídák's testing to correct for multiple comparisons, only WT, H98D and H100D displayed here **(D)** – **(F)** *In silico* docking results of *At*PCO4 WT and His-triad variants (based on PDB 6S7E and created using the mutagenesis tool in PyMOL) with RAP2.12_2-6_ using HADDOCK2.4. The predicted models are presented as cartoon and other active site residues investigated are indicated as sticks and with bold letters. Modelled complexes imply monodentate coordination of peptide to active site metal, however recent experimental evidence in ADO suggests bidentate coordination is more likely(10, 11). Docking constraints may bias our models and the denticity of substrate binding in PCOs remains to be determined.

Figure S4. *In silico* docking models of ERF-VII and non-ERF-VII substrates to *At*PCO4-2 WT using Haddock2.1.

Cartoon representation of the active site of *At*PCO4-2 (PDB: 6S7E) WT with its 5-mer peptide substrates **(A)** RAP2.12_2–6_, **(B)** and **(C)** ZPR2_2-6_ and **(D)** VRN2_2-7_ docked using HADDOCK 2.4.(6) **(B)** and **(C)** Two different clusters of the docked ZPR2_2-6_ substrate. **(E)** Overlay of the docked RAP2.12_2–6_, ZPR2_2-6_ and VRN2_2-7_ substrates in the active site of *At*PCO4-2 WT. ZPR2_2-6_ and VRN2_2-7_ are peptides representing the Cys2-initiating N-terminal sequences of known PCO substrates little zipper 2 and vernalisation 2.(4, 7–9) Modelled complexes imply monodentate coordination of peptide to active site metal, however recent experimental evidence in ADO suggests bidentate coordination is more likely(10, 11). Docking constraints may bias our models and the denticity of substrate binding in PCOs remains to be determined.

Figure S5. Substrate binding of *Hs*ADO, *At*PCO4-2 and *Hs*CDO-1.

Cartoon representation of the active site of **(A)** *Hs*ADO (PDB: 7REI) with its 4-mer peptide substrate RGS5_2–5_ and **(B)** *At*PCO4-2 (PDB: 6S7E) with its 5-mer peptide substrate RAP2.12_2–6_ docked using HADDOCK 2.4.(6) Modelled complexes imply monodentate coordination of peptide to active site metal, however recent experimental evidence in ADO suggests bidentate coordination is more likely(10, 11). Docking constraints may bias our models and the denticity of substrate binding in PCOs remains to be determined. **(C)** Cartoon representation of the active site of *Hs*CDO-1 with its substrate cysteine (PDB: 6BGF) positioned in the crystal structure. Key residues and substrates are represented as sticks. Active site metals are represented as green (Ni) or orange (Fe) spheres. Dashed lines represent distances between atoms (Å).

Figure S6. Overview of recombinant purified *At*PCO4 WT and variants.

**(A)** Cartoon representation of the *At*PCO4 active site (PDB: 6S7E). **(B)** Left, SDS-PAGE gel of *At*PCO4 WT and active site variants after storage at - 80 °C and, on the right, of a SEC fraction from *At*PCO4 H98D stained with QuickBlue Protein Stain.

Figure S7. CD analysis of active site variants.

**(A)** Cartoon representation of the *At*PCO4 active site (PDB: 6S7E). **(B)** average CD spectra of *At*PCO4 enzymes (n=3) after subtracting the baseline and converting millidegrees into Δε using BeStSel^TM^ (Beta Structure Selection) webserver. The CD spectra were measured at a protein concentration of 0.2 mg.mL^-1^ (~ 7.3 μM, 245 residues) in 10 mM KH_2_PO_4_ (pH 7.5), 0.1 cm pathlength, 260 nm monochromator wavelength, 1.0 nm bandwidth, 185 – 250 nm in 0.5 nm steps, 0.5 s time-per-point and at 25 °C. Sample of variant H100D contained a high salt concentration (400 mM NaCl) known to interfere with the detected absorbance below 200 – 205 nm. The control WT sample for H100D (WT for H100D) was therefore also measured with 400 mM NaCl. **(C)** Predictions of *At*PCO4 enzyme secondary structures from crystal structure of His_6_-tagged *At*PCO4-1 (PDB: 6S7E) and experimental CD results of His_6_-cleaved *At*PCO4-2 using BeStSel^TM^. RMSD: root mean square standard deviation, calculated by BeStSel^TM^ from experimental vs fitted spectra; NRMSD: normalised RMSD, calculated by BeStSel^TM^ from smoothed experimental vs fitted spectra. PDBMD2CD, DSSP (define secondary structure of proteins) and SELCON (self-consistent method) were also used to interpret proportions of secondary structure from the 6S7E crystal structure.

Figure S8. CD spectra of iron removed *At*PCO4 enzymes supplemented with nickel.

**(A)** Cartoon representation of the *At*PCO4 active site (PDB: 6S7E). **(B)** CD spectra of iron removed and with nickel-substituted (2:1 Ni to protein, as used in binding assays) *At*PCO4 enzymes after subtracting the baseline (n=3) after subtracting the baseline and converting millidegrees into Δε using BeStSel^TM^ (Beta Structure Selection) webserver. The CD spectra were measured at a protein concentration of 0.2 mg.mL^-1^ (~ 7.3 μM, 245 residues) in 10 mM KH_2_PO_4_ (pH 7.5), 0.1 cm pathlength, 260 nm monochromator wavelength, 1.0 nm bandwidth, 185 – 250 nm in 0.5 nm steps, 0.5 s time-per-point and at 25 °C. **(C)** Predictions of *At*PCO4 enzyme secondary structures from experimental CD results of His-cleaved *At*PCO4-2 using BeStSel^TM^. **(D)** Iron occupancy of *At*PCO4 WT after removing the iron during the purification process determined by ICP-MS (technical replicates, n = 2). Error bars indicate the ± accuracy error. RMSD: root mean square standard deviation, calculated by BeStSel^TM^ from experimental vs fitted spectra; NRMSD: normalised RMSD, calculated by BeStSel^TM^ from smoothed experimental vs fitted spectra.

Figure S9. Optimisation of supplemental iron for *At*PCO4-2 wt and variants. The activities of *At*PCO4-2 enzymes (0.1 μM) towards 500 μM RAP2.12_2-15_ in reaction buffer (50 mM bis-tris propane, 50 mM NaCl, pH 8) supplemented with 1 mM l-ascorbate, 5 mM TCEP (pH 8) and iron concentrations ranging from 0 – 200 μM. RAP2.12_2-15_ oxidation was measured after 10 minutes (except Y183F, for which RAP2.12_2-15_ oxidation was measured after 5 minutes); data obtained in technical triplicates (n = 3). 50 µM Fe(II) was selected for supplementation of WT and all variants except S107L (100 µM) and Y183F (75 µM).

Figure S10. Iron occupancy and O_2_ sensitivity of variants with mutations at the substrate entry site.

**(A)** Cartoon representation of *At*PCO4 active site obtained from a crystal structure (PDB: 6S7E) with substrate entry site residues highlighted in bright blue. **(B)** Iron occupancy determined using ICP-MS (technical replicates, n = 2). Statistical significance of all active site variants compared to WT was determined using one-way ANOVA and Holm-Šídák's testing to correct for multiple comparisons, only WT, Y73R and Y183F displayed here (GraphPad Prism). **(C)** Initial velocities of *At*PCO4 Y183F (in technical triplicates, n = 3) from 0 – 2 min time courses plotted against RAP2_2-15_ peptide substrate concentrations. Solid line is the fit of the Michaelis-Menten equation to the data. Standard deviation is indicated by error bars. **(D)** O_2_ sensitivity of AtPCO4 WT and variant Y183F measured after 1 min reaction time (left, technical replicates: WT 2 x n = 3, Y183F n = 3). Standard deviation is indicated by error bars. Michaelis-Menten constants were estimated using GraphPad Prism with error bars indicating the standard error of the mean. Statistical significance could not be evaluated because the Michaelis-Menten model was not applicable (N/A) for variant Y183F.

Figure S11. Substrate binding and iron occupancy of variants with mutations *trans* to His-triad.

**(A)** Cartoon representation of *At*PCO4 active site obtained from a crystal structure (PDB: 6S7E). Residues that are positioned *trans* to the His-triad are highlighted in green. **(B)** Iron occupancy determined using ICP-MS (technical replicates, n = 2). Statistical significance of all active site variants compared to WT was determined using one-way ANOVA and Holm-Šídák's testing to correct for multiple comparisons, only WT, V105G, S107L and D177E displayed here (****: P < 0.0001, GraphPad Prism). **(C)** and **(D)** *in silico* docking results of *At*PCO4 WT and variants (based on PDB 6S7E and created using the mutagenesis tool in PyMOL) with RAP2.12_2-8_ using HADDOCK2.4.(6, 12) The predicted variant models (in panel **(C)** for variant V105G, in panel **(D)** cluster1_3 and cluster1_2 for variant D177E) were aligned with the WT model and are presented as cartoon. Other investigated active site residues are indicated in bold letters. Modelled complexes imply monodentate coordination of peptide to active site metal, however recent experimental evidence in ADO suggests bidentate coordination is more likely(10, 11). Docking constraints may bias our models and the denticity of substrate binding in PCOs remains to be determined. **(D)** The docking models for variant D177E indicate that E177 can either coordinate the iron (cluster1_3) or still coordinate the substrate (cluster1_2).

Figure S12. Substrate binding and iron occupancy of variants with mutations *trans* to the substrate entry site.

**(A)** and **(B)** show structures of *in silico* docking results of *At*PCO4 WT and variant C173A (based on PDB 6S7E and created using the mutagenesis tool in PyMOL) with RAP2.12_2-8_ predicted using HADDOCK2.4(6, 12) and presented as cartoons. Other investigated active site residues are indicated as sticks and in **(A)** highlighted in bold letters. Modelled complexes imply monodentate coordination of peptide to active site metal, however recent experimental evidence in ADO suggests bidentate coordination is more likely(10, 11). Docking constraints may bias our models and the denticity of substrate binding in PCOs remains to be determined. **(B)** Distance of side chains from residues C173 and I175 to the Nt-Cys are indicated in dashes with values presented in Å. **(C)** Iron occupancy determined using ICP-MS (technical replicates, n = 2). Statistical significance of all active site variants compared to WT was determined using one-way ANOVA and Holm-Šídák's testing to correct for multiple comparisons, only WT, C173A and I175F displayed here (****: P < 0.0001, GraphPad Prism). **(D)** Comparison of enzyme activity between plate-based assays and O_2_ assays for which vials were used to enable measurements at specific O_2_ concentrations. The activity of 0.1 μM *At*PCO4 WT and variant C173A (technical replicates, n = 3, n = 5 for O_2_ assay) was measured with buffer, substrate and enzyme volumes and incubation timings used in the different assay setups at optimal reaction conditions (RAP2.12_2–15_, iron, l-ascorbate) after 1 min in assay buffer (50 mM bis-tris-propane, pH 8, 50 mM NaCl) with 5 mM TCEP at 21% O_2_. **(E)** and **(F)** dissociation curves of RAP2.12_2-15_ from *At*PCO4 WT (n = 6) and variant C173A (n = 3) acquired using changes in tryptophan fluorescence intensity (TFI). A specific binding model with Hill slope (Y = *B_max_**X^h^*(*K_d_^h^*+X^h^)^-1^) was fitted to the data. Error bars indicate the standard error (GraphPad Prism).

Figure S13. Complementation of *4pco* Arabidopsis plants with *AtPCO4* *WT*, *C173A* and *Y183F*.

Genotyping results of *4pco* knockout plants complemented with *AtPCO4* *WT* and mutants. Amplified PCR products that correspond to the *AtPCO4* insert are shown in the top panel. Plants homozygous for *AtPCO5*^-/-^ were identified by an amplified PCR product for the T-DNA insert (bottom panel) and lack of amplification for *AtPCO5 WT* (middle panel). Lanes containing PCR products from plant lines and controls are indicated.

Figure S14. Sequencing results from complemented lines wt-1 and -2*.*

DNA sequences were determined by sequencing of PCR products from Arabidopsis lines wt-1 and wt-2 and translated using SnapGene Viewer (Version 6.0.7). The wildtype *At*PCO4-2 sequence is shown on the top in bold and the experimentally obtained sequences were aligned using Clustal Omega(13) and named after their plant line followed by the primer name used for sequencing.

Figure S15. Sequencing results from complemented lines C173A-1 and -2*.*

DNA sequences were determined by sequencing of PCR products from Arabidopsis lines C173A-1 and C173A-2 and translated using SnapGene Viewer (Version 6.0.7). The wildtype *At*PCO4-2 sequence is shown on the top in bold and the experimentally obtained sequences were aligned using Clustal Omega(13) and named after their plant line followed by the primer name used for sequencing. The mutated residue C173A is highlighted in a red box.

Figure S16. Sequencing results from complemented line Y183F*.*

DNA sequences were determined by sequencing of PCR products from Arabidopsis line Y183F and translated using SnapGene Viewer (Version 6.0.7). The wildtype *At*PCO4-2 sequence is shown on the top in bold and the experimentally obtained sequences were aligned using Clustal Omega(13) and named after their plant line followed by the primer name used for sequencing. The mutated residue Y183F is highlighted in a red box.

Figure S17. Phenotype of *4pco AtPCO4* complemented lines.

**(A)** Germinated seeds at day 2. **(B)** Germination rate of around 24 seeds per line, i.e., 26 seeds for Col-0, 25 seeds for wt-1, 26 seeds for wt-2, 27 seeds for C173A-1, 30 seeds for C173A-2 and 38 seeds for Y183F sown on agar plates (½ MS, 1% sucrose, 0.8% agar, pH 5.7) and incubated with 16 h photoperiod per 24 h. **(C)** Plants grown on submergence compost (1:1 modular seed and John Innes No.2 with ¼ fine vermiculite) until 3 weeks. Plants were used for submergence experiment at 9-leaf stage. **(D)** Leaf perimeter, leaf canopy size and total leaf number were analysed from > 7 plants / line from day 7 post-sowing using the open-source software Leaf-GP(5) (values in Tables S6 – S8). Values were excluded, when the software could not assess the photo of a plant appropriately, e.g., rosette too small or mixture of multiple plants considered as one.

Figure S18. *AtPCO4* and *5* transcript levels of 5.5-week-old *4pco AtPCO4* plants.

RT-qPCR results were normalised to transcript levels of the housekeeping gene UBQ10 and Col-0. **(A)** Transcript levels of inserted *AtPCO4* were determined using three different primer sets: **(C)** PCO4_KO_1, **(D)** qpAtPCO4-2 and **(E)** sgPCO4. The latter two primer sets covered a similar region +/- 2 bp. Transformed *AtPCO4* transcript levels were similar between all primer sets except for line wt-1 for which either no transcript levels (PCO4_KO_1) similar to Col-0 or reduced levels were determined (qpAtPCO4-2, sgPCO4). **(B)** *AtPCO5* transcript levels were measured using primer set AtPCO5_1 to confirm *pco5^-/-^* and therefore the *4pco* genotype. **(B)** and **(E)** show data from two independent experiments with two technical replicates of four biological samples each (n = 2 x (2 x 4); circle: 1^st^ experiment, triangle: 2^nd^ experiment). Statistical significance (P < 0.05) was obtained using one-way ANOVA and the Holm-Šídák's test for multiple comparison testing (GraphPad Prism). **(C)** and **(D)** show data from four biological samples with two technical replicates each (n = 2 x 4). Statistical significance (P < 0.05) was obtained using one-way ANOVA and the Holm-Šídák's test for multiple comparison testing (GraphPad Prism).

Figure S19. Statistical significance of differences in HRG transcript levels of *4pco AtPCO4* plants.

Change in transcript levels of HRGs in 5.5-week-old *4pco AtPCO4* and Col-0 plants. Two independent experiments with two technical replicates of four biological samples each (n = 2 x (2 x 4); circle: 1^st^ experiment, triangle: 2^nd^ experiment). Statistical significance (P < 0.05) was obtained using one-way ANOVA and the Holm-Šídák's test for multiple comparison testing (GraphPad Prism).

Figure S20. Submergence recovery of all *4pco* lines and their Col-0 control plants.

Photos of all submerged plants after 10 days of recovery. Each tray contains a complemented *4pco* line and Col-0 as control. For line Y183F, only eight plants were available for submergence in the first experiment which were submerged for 3.5 days.

Figure S21. Fresh and dry weight of *4pco AtPCO4* *WT*, *C173A*, *Y183F* and Col-0 control plants after 2.5 and 3.5 d submergence.

Fresh and dry weight of plants submerged for **(A)** – **(D)** 2.5 and **(E)** – **(H)** 3.5 days with **(A, C, E, G)** and without **(B, D, F, H)** dark control group after 10 days of recovery (lower case letters in legends indicate plants submerged in the same box). Dead plants were considered to weigh 0 g. The statistical significance (P < 0.05) was determined using two-way ANOVA followed by Tukey's multiple comparisons test (GraphPad Prism).

**Supplementary Discussion**

**Additional discussion of the kinetic properties and predicted structural properties of *At*PCO4 variants**

**Fe(II)-binding Residues: H98 and H100**

Our results confirmed that the His-triad is essential for *At*PCO4 activity as replacing H98 or H100 with an Asp diminished *At*PCO4 activity, to trace levels, similar to reported results for *At*PCO4-1 for H164D(1) and *At*PCO5 for H165(14). *In silico* docking models of *At*PCO4 H98D and H100D (Figure S3) predicted a distorted iron coordination for both variants which potentially caused the reduced iron binding capacity and the depleted catalytic activity of these variants. Previous density functional theory studies of the thiol dioxygenase CDO predicted that mutating His-triad residues to Asp in CDO could alter O_2_ activation and its transfer onto the Cys-thiol,(15) which may be consistent with the distorted iron coordination seen in these *At*PCO4 variants, H98D and H100D.

**Substrate-binding Residues: Y73 and Y183**

Mutating the substrate entry site residue Y73R diminished the activity of *At*PCO4 almost completely (Figure 2). However, this correlated with a high *K_d_* and thus was highly likely due to reduced substrate affinity, suggesting that Y73 is involved in substrate binding. The respective Tyr in *Hs*ADO, Y87, was proposed to affect *Hs*ADO activity by modifying the positioning of the D206-carboxylate (equivalent residue in *At*PCO4: D177) via an H-bond network.(10) In *Hs*ADO, mutations Y87A and Y87F did not impact substrate binding (*K_d_*) considerably(10) suggesting that mutation Y73R in *At*PCO4 decreased substrate affinity mostly due to the change to a positively charged / basic side chain (rather than loss of the benzene ring or hydroxyl group). The other residue at the proposed substrate entry site, Y183, was mutated to a Phe as examined previously by White *et al.*, 2020 in *At*PCO4-1(1) and by Chen *et al.*, 2021 in *At*PCO5(14). The mutation Y183F in *At*PCO4 showed a similar reduction in activity as the previous results from *At*PCO4-1 and *At*PCO5 which can now be assigned to the decreased substrate affinity of the Y183F variant to RAP2.12_2-15_. These findings correlate with the recent study of *Hs*ADO in which the authors mutated the respective Tyr residue to an Ala and Phe.(10) Both variants, *Hs*ADO Y212A and Y212F, were less active due to reduced substrate affinity.(10) This Tyr residue in *Hs*ADO is reported to be involved in a H-bond network with a water molecule and the Nt-Ser-OH of a cyclic peptide inhibitor (PDB: 9DXB). An *At*PCO2 model with Cys as substrate reported by Chen *et al*., 2021(14) proposed that the Tyr at the equivalent position as Y183 in *At*PCO4 forms a H-bond with the Nt-Cys-NH_3_^+^. Both predictions for Nt-Cys coordination in the active site suggest an Nt-Cys stabilising function of *At*PCO4 Y183 which would correlate with the decrease in substrate affinity *in vitro* that we observe when the Tyr-OH is removed. It remains to be determined if the Nt-Cys-SH or -NH_3_^+^ is stabilised by *At*PCO4 Y183.

**Residues trans to the His-triad: Asp177, Val105 and Ser107**

It has been proposed that the conserved active site Asp residue in NCOs is also involved in stabilising the Nt-Cys. In the *At*PCO2-Cys model(14), the small molecule substrate Cys-thiol is predicted to form an H-bond with the *At*PCO2 D177-carboxyl group (*At*PCO4 numbering), whereas in the structure of *Hs*ADO complexed with a cyclic peptide inhibitor, the substrate’s Nt-amine group interacts with the equivalent Asp-carboxyl via an H-bond (PDB: 9DXB). In addition, overlaying the *At*PCO4 structure with the *Hs*ADO cyclic peptide inhibitor structure suggests that D177 is in a similar position to the equivalent Asp in *Hs*ADO; thus any role this Asp residue may play in interacting with oxygen or reaction intermediates, as proposed by Jiramongkol *et al.*(10), could also be taking place in *At*PCO4. These structural insights and predictions support the kinetic data showing that residue D177 is important for catalysis (Figure 3). With the D177E variant, extending the residue (by one methylene group) while keeping the charge state, ablated enzymatic activity by reducing the catalytic rate. However, this mutant can still facilitate substrate binding with an affinity similar to the WT enzyme (Figure 3G). These results were in line with data from *Hs*ADO variants of the equivalent Asp residue.(10) Mutating the Asp to an Asn, as reported previously for *At*PCO4(1), could result in a weaker H-bond between the Asn-amide and Nt-Cys substrate than with an Asp-carboxylate, potentially further reducing catalytic activity, which has been observed when comparing both mutations in *Hs*ADO(10). For ADO, a model in which D206 is facing the Nt-Cys-amine seems to be favoured, both because it has been observed to be in H-bonding distance of the Nt-Ser-amine of a cyclic peptide inhibitor in a crystal complex structure (PDB: 9DXB)(10) and because it has lowest calculated energetic states in computational models (11, 16).

The other two mutations *trans* to the iron-coordinating His-triad, V105G and S107L, also impacted the catalytic rate and catalytic efficiency of *At*PCO4 (Figure 3). Variant S107L appeared to have a lower catalytic rate and catalytic efficiency due to its reduced iron binding capacity and, potentially, modified positioning of the co-substrate O_2_, reaction intermediate and/or the *At*PCO4 D177-carboxylate. Residue S107 could interact with the D177-carboxylate via an H-bond pulling the side chain into the active site and potentially towards the O_2_ co-substrate. *Hs*ADO showed reduced activity when the equivalent Asp side chain (D206 in *Hs*ADO, D177 in *At*PCO4) was pulled towards the substrate entry site (PDB: 9DXU), i.e., in the opposite direction of *At*PCO4 S107, due to a cyclic peptide inhibitor(10). However, the complete catalytic mechanism might be slightly different in *Hs*ADO than in *At*PCO4 because in *At*PCO4 the Ser at position 107 is replaced by a Leu in *Hs*ADO while the residue spatially below is a Val in *At*PCO4 but a Gly in *Hs*ADO (Figure 1B). These residues, Ser and Val in *At*PCO4 and Leu and Gly in *Hs*ADO, might affect the catalytic mechanism in both NCO enzymes by optimising the position for molecular O_2_ coming into the active site in each enzyme (water molecules in *Hs*ADO:cyclic peptide structure PDB: 9DXB) and of the Asp residue that coordinates the protein and O_2_ substrate. Reducing the size of S107 in *At*PCO4 in a previous study, S107A, did not affect *At*PCO4 activity(1) suggesting that a smaller but not larger residue is allowed at this position. Reducing the size of residue V105 with variant V105G, only affected the *V_max_* and catalytic rate and thereby the catalytic efficiency of *At*PCO4 but did not affect peptide substrate binding affinity (Figure 3). This further supported the hypothesis that residue V105 might be involved in optimising the position of the O_2_ substrate in the active site by pushing it towards residues S107 and D177 by limiting the space elsewhere. The effect of space limitation in the active site was also observed for ADO and its small molecule substrate cysteamine; ADO activity could be increased by increasing glycerol concentrations which led to a transition from monodentate to bidentate coordination.(11)

**Residues trans to the substrate entry site: C173 and I175**

Mutating a residue *trans* to the substrate entry site, C173 to A173, appeared to affect the catalytic rate of *At*PCO4 once O_2_ was coordinated in the active site (Figure 4). The other residue *trans* to the substrate entry site that was investigated, I175 mutated to F175, showed RAP2.12_2-15_ kinetics similar to variant C173A (Figure 4). The results supported the hypothesis that residues C173 and I175 are located closely to O_2_ and/or reaction intermediates, and thus can affect O_2_ activation and/or oxygen transfer onto the Nt-Cys-thiol. Examination of equivalent residues in *Hs*ADO in the recently reported crystal structure with bound cyclic peptide (PDB: 9DXB) would suggest that both residues are likely to be too far away from the reaction centre to be involved in substrate binding. Any effects of the mutations might therefore be due to impacts on the *At*PCO4 structure or constrictions on conformational changes necessary for efficient catalysis.

**References**

1.	White, M. D., Dalle Carbonare, L., Lavilla Puerta, M., Iacopino, S., Edwards, M., Dunne, K., Pires, E., Levy, C., McDonough, M. A., Licausi, F., and Flashman, E. (2020) Structures of Arabidopsis thaliana oxygen-sensing plant cysteine oxidases 4 and 5 enable targeted manipulation of their activity. *Proceedings of the National Academy of Sciences*. **117**, 23140–23147

2.	Masson, N., Keeley, T. P., Giuntoli, B., White, M. D., Puerta, M. L., Perata, P., Hopkinson, R. J., Flashman, E., Licausi, F., and Ratcliffe, P. J. (2019) Conserved N-terminal cysteine dioxygenases transduce responses to hypoxia in animals and plants. *Science (1979).* **365**, 65–69

3.	Licausi, F., Kosmacz, M., Weits, D. A., Giuntoli, B., Giorgi, F. M., Voesenek, L. A. C. J., Perata, P., and van Dongen, J. T. (2011) Oxygen sensing in plants is mediated by an N-end rule pathway for protein destabilization. *Nature*. **479**, 419–422

4.	Gibbs, D. J., Tedds, H. M., Labandera, A.-M., Bailey, M., White, M. D., Hartman, S., Sprigg, C., Mogg, S. L., Osborne, R., Dambire, C., Boeckx, T., Paling, Z., Voesenek, L. A. C. J., Flashman, E., and Holdsworth, M. J. (2018) Oxygen-dependent proteolysis regulates the stability of angiosperm polycomb repressive complex 2 subunit VERNALIZATION 2. *Nat. Commun.* **9**, 5438

5.	Zhou, J., Applegate, C., Alonso, A. D., Reynolds, D., Orford, S., Mackiewicz, M., Griffiths, S., Penfield, S., and Pullen, N. (2017) Leaf-GP: an open and automated software application for measuring growth phenotypes for arabidopsis and wheat. *Plant Methods*. **13**, 117

6.	Honorato, R. V., Koukos, P. I., Jiménez-García, B., Tsaregorodtsev, A., Verlato, M., Giachetti, A., Rosato, A., and Bonvin, A. M. J. J. (2021) Structural Biology in the Clouds: The WeNMR-EOSC Ecosystem. *Front. Mol. Biosci.* **8**, 729513

7.	Weits, D. A., Kunkowska, A. B., Kamps, N. C. W., Portz, K. M. S., Packbier, N. K., Nemec Venza, Z., Gaillochet, C., Lohmann, J. U., Pedersen, O., van Dongen, J. T., and Licausi, F. (2019) An apical hypoxic niche sets the pace of shoot meristem activity. *Nature*. **569**, 714–717

8.	Labandera, A., Tedds, H. M., Bailey, M., Sprigg, C., Etherington, R. D., Akintewe, O., Kalleechurn, G., Holdsworth, M. J., and Gibbs, D. J. (2021) The PRT6 N‐degron pathway restricts VERNALIZATION 2 to endogenous hypoxic niches to modulate plant development. *New Phytologist*. **229**, 126–139

9.	Gunawardana, D. M., Southern, D. A., and Flashman, E. (2024) Measuring plant cysteine oxidase interactions with substrates using intrinsic tryptophan fluorescence. *Sci. Rep.* **14**, 1–10

10.	Jiramongkol, Y., Patel, K., Johansen-Leete, J., Maxwell, J. W. C., Chang, Y., Du, J. J., Passioura, T., Cook, K. M., Payne, R. J., and White, M. D. (2025) An mRNA-display derived cyclic peptide scaffold reveals the substrate binding interactions of an N-terminal cysteine oxidase. *Nat. Commun.* **16**, 4761

11.	Helms, J. R., Probst, M., Paris, J., Szamweber, P., Zhao, Z., Wu, S., and Pierce, B. S. (2026) EPR spectroscopy reveals glycerol-dependent activation of cysteamine dioxygenase (ADO) enables bidentate substrate coordination. *Journal of Biological Chemistry*. 10.1016/j.jbc.2026.111438

12.	Van Zundert, G. C. P., Rodrigues, J. P. G. L. M., Trellet, M., Schmitz, C., Kastritis, P. L., Karaca, E., Melquiond, A. S. J., Van Dijk, M., De Vries, S. J., and Bonvin, A. M. J. J. (2016) The HADDOCK2.2 Web Server: User-Friendly Integrative Modeling of Biomolecular Complexes. *J. Mol. Biol.* **428**, 720–725

13.	Madeira, F., Pearce, M., Tivey, A. R. N., Basutkar, P., Lee, J., Edbali, O., Madhusoodanan, N., Kolesnikov, A., and Lopez, R. (2022) Search and sequence analysis tools services from EMBL-EBI in 2022. *Nucleic Acids Res.* **50**, W276–W279

14.	Chen, Z., Guo, Q., Wu, G., Wen, J., Liao, S., and Xu, C. (2021) Molecular basis for cysteine oxidation by plant cysteine oxidases from *Arabidopsis thaliana*. *J. Struct. Biol.* **213**, 107663

15.	de Visser, S. P., and Straganz, G. D. (2009) Why Do Cysteine Dioxygenase Enzymes Contain a 3-His Ligand Motif Rather than a 2His/1Asp Motif Like Most Nonheme Dioxygenases? *J. Phys. Chem. A*. **113**, 1835–1846

16.	Fernandez, R. L., Elmendorf, L. D., Smith, R. W., Bingman, C. A., Fox, B. G., and Brunold, T. C. (2021) The Crystal Structure of Cysteamine Dioxygenase Reveals the Origin of the Large Substrate Scope of This Vital Mammalian Enzyme. *Biochemistry*. **60**, 3728–3737
